# Bacterial Strains from Soybean Nodules in the Lower Volga Region Belong to a New Subspecies *Bradyrhizobium japonicum* subsp. *saratovii* subsp. nov.

**DOI:** 10.3390/microorganisms14030684

**Published:** 2026-03-18

**Authors:** Aleksandr S. Sidorin, Gennady L. Burygin, Andrey V. Fedorov, Aleksandr D. Katyshev, Yaroslav M. Krasnov, Oksana V. Tkachenko

**Affiliations:** 1 Department of Plant Breeding, Selection, and Genetics, Institute of Genetics and Agronomy, Saratov State University of Genetics, Biotechnology and Engineering Named After N.I. Vavilov, Saratov 410012, Russia; sasha.sidorin2013@mail.ru (A.S.S.); oktkachenko@yandex.ru (O.V.T.); 2 Russian Research Anti-Plague Institute “Microbe”, Saratov 410005, Russia; andl4@yandex.ru (A.V.F.); katishevs10@gmail.com (A.D.K.); yarkras@gmail.com (Y.M.K.); 3 Institute of Biochemistry and Physiology of Plants and Microorganisms, Saratov Scientific Centre of the Russian Academy of Sciences, Saratov 410049, Russia; 4 Department of Organic and Bioorganic Chemistry, Institute of Chemistry, Saratov State University, Saratov 410012, Russia

**Keywords:** *Bradyrhizobium*, soybean, Lower Volga region, genome-based taxonomy, subspecies

## Abstract

The isolation of locally adapted rhizobial strains with high symbiotic activity represents an effective strategy for increasing soybean yield under extreme environmental conditions. In this study, seven novel strains were isolated from nodules of soybeans grown in a greenhouse using field soil from the Lower Volga region. Five genomes were assembled into complete circular chromosomes, whereas two strains yielded near-complete chromosomes containing single repeat-mediated junctions. All strains had putative plasmids that were independently validated as circular by long-read mapping and confirmed by the presence of characteristic replication and conjugation-associated genes. Genome sequences of strains were about 11 Mb, and GC contents were 63.1–63.3%. Comparative genome analyses demonstrated that all strains had average nucleotide identity values of 95.4% with *Bradyrhizobium japonicum* USDA 6^T^ and 96.3% with *Bradyrhizobium barranii* 144S4^T^, forming a distinct cluster in phylogenetic trees. No significant differences were detected between *B. japonicum* and *B. barranii* that would explain the species boundary. Therefore, it is proposed to unite all novel strains into the subspecies *Bradyrhizobium japonicum* subsp. *saratovii* subsp. nov., and all other strains of *B. japonicum* and *B. barranii* we suggest dividing into four subspecies: *Bradyrhizobium japonicum* subsp. *japonicum* subsp. nov., *Bradyrhizobium japonicum* subsp. *barranii* comb. nov., *Bradyrhizobium japonicum* subsp. *apii* comb. nov., and *Bradyrhizobium japonicum* subsp. *saratovii* subsp. nov. The proposed taxonomic framework expands current knowledge of the biodiversity of soybean symbiotic bacteria and contributes to a better understanding of the distribution and the evolution of bacteria *Bradyrhizobium* spp. in previously unexplored regions.

## 1. Introduction

In modern agrobiotechnology, microbial biologics have gained increasing popularity as a means of rational soil resource management [1,2,3,4,5]. Biological fertilizers typically contain symbiotic nitrogen-fixing bacteria [6,7]. However, not all strains included in currently used bioproducts are capable of exerting an effective plant growth-promoting effect. This is due to their high host specificity, low persistence on plant roots, and poor adaptation to the soil and climatic conditions of the target region [8]. An effective solution to this problem is the isolation and selection of efficient indigenous bacterial strains from the territory where crop cultivation is planned. Strains obtained in this way are already adapted to the local edaphic and climatic conditions.

Leguminous crops are characterized by high economic and ecological importance for agricultural production [9]. Soybean (*Glycine max* (L.) Merr.), as one of the most important representatives of this plant family, is of particular interest due to its high protein and oil content in the grain. In 2024, soybeans were cultivated worldwide on approximately 143 million ha, with a total production of 398 million tons [10]. The most important soybean producers are Brazil and the United States, which together account for about 66% of global production. However, annual production exceeds 1 million tons in at least 12 other countries. Over the past decade, global soybean production has increased by 23%, which is associated with improved production efficiency and an 18.5% expansion of the cultivated area. Russia ranks eighth among the world’s soybean producers, with a production of 7.0 million tons harvested from 4.13 million ha. Moreover, the area under soybean cultivation in Russia has been steadily increasing and has expanded by 98% since 2015. Consequently, soybean cultivation is spreading into large areas where this crop was previously not grown.

The productivity of soybeans, as well as other leguminous crops, largely depends on the formation of symbioses with root nodule bacteria capable of fixing atmospheric nitrogen [11]. When isolating bacteria from soybean nodules, strains belonging to the genera *Bradyrhizobium*, *Mesorhizobium*, *Agrobacterium*, *Sinorhizobium*, and others are most frequently obtained [12,13,14]. Among these, bacteria of the genus *Bradyrhizobium* are the most widely used as inoculants [15,16,17,18,19,20]. Since the use of rhizobial strains as biofertilizers underlies modern soybean cultivation technologies, it is essential to evaluate the ability of potential inoculants to form effective symbioses with plants under new ecological conditions.

The Lower Volga region (Russia), including the Saratov region, is a non-traditional region for soybean cultivation. Its soil and climatic conditions differ markedly from those of conventional soybean-growing regions, primarily due to low rainfall. As a result, soybeans are cultivated mainly under irrigation in this region, and the cultivated area has been increasing rapidly in recent years. Because soybean is an introduced crop in the Volga region, local soils lack soybean-specific symbiotic bacteria, while commercial *Bradyrhizobium* spp. strains used in biofertilizers are exposed to non-optimal environmental conditions.

Bacteria of the genus *Bradyrhizobium* belong to the class *Alphaproteobacteria*, family *Nitrobacteraceae* (order *Hyphomicrobiales*) [21]. The genus currently comprises 75 validly published and 19 non-validly published species [22]. The large number of species within the genus makes it difficult to assign new strains of *Bradyrhizobium* spp. to a particular species. Standard 16S rRNA testing is not applicable to this genus due to the high percentage of sequence identity between representatives of different species [23]. For species identification of strains, whole-genome sequence analysis is used. The average nucleotide identity (ANI), for which it is proposed to use 94–96.5% and 97–98% as the levels of species and subspecies boundries, respectively: ANI values of less than 94% for strains of different species, 95–97% for strains of the same species but different subspecies, and more than 98% for strains of the same subspecies [24,25,26].

Many strains of *Bradyrhizobium* spp. are of considerable economic importance due to their ability to fix atmospheric nitrogen and form effective symbioses with leguminous crops [27,28,29]. There are no published reports describing the isolation of *Bradyrhizobium* root nodule bacteria from the Lower Volga region, a newly explored area for soybean cultivation. The aim of the present study was to isolate bacteria from nodules of soybeans grown in a greenhouse using field soil from the Lower Volga region, to characterize them, and to perform their taxonomic identification.

## 2. Materials and Methods

### 2.1. Bacterial Strains and Culture Conditions

Root nodules of natural origin were collected from soybean plants (*Glycine max* (L.) Merr.), cultivar Natalie, grown in a greenhouse in soil sampled from the educational and experimental farm “Povolzhye” of Vavilov University in 2023 (Saratov region, Russia; 51.126105146540645, 45.999733721150704). The soil cover of the farm is represented by typical dark chestnut soils (Kastanozem, WRB). The soil texture corresponds to medium and heavy loams. The humus content in the 0–30 cm soil layer was 2.9%. The soil was slightly alkaline (pH 7.2) and characterized by low nitrate nitrogen availability (6 mg/kg), a moderate content of available phosphorus (16.3 mg/kg), and a high level of exchangeable potassium (300 mg/kg). Maize (*Zea mays* L.) was used as the preceding crop.

Soybean plants were cultivated in the greenhouse for 30 days in 3-L vessels with field soil from the farm “Povolzhye” and, at the time of bacterial isolation, were at the early flowering stage. Nodules were formed on the main root and on first-order lateral roots. The nodules selected for the study were thoroughly washed with sterile distilled water to remove adhering soil particles. To eliminate epiphytic microorganisms, soybean nodules were sequentially treated with a 1% sodium hypochlorite solution for 2 min and 70% ethanol for 30 s. Surface-sterilized nodules were homogenized in a sterile mortar with the addition of 1–2 mL of physiological saline. A series of tenfold dilutions was prepared from the resulting homogenate, and 10 µL of each dilution was plated onto solid Yeast Mannitol Agar (YMA) medium of the following composition (g/L): yeast extract, 0.5; K_2_HPO_4_, 0.2; MgSO_4_·7H_2_O, 0.2; mannitol, 7.0; agar, 15 (pH 7.0–7.2) [30]. Plates were incubated for 7–10 days at 28 °C until colonies appeared. Pure cultures were obtained by repeated culturing of single colonies. Cultures were maintained on solid YMA at 4 °C and at −20 °C in liquid YMA supplemented with 15% glycerol. A total of ten strains of root nodule bacteria were isolated.

The ability of the strains to form functional nodules on plants was evaluated by reinoculation of soybean seeds (cv. Natalie). Seeds were sterilized in 0.2% diacid solution for 2 min, then washed with sterile distilled water on a magnetic stirrer five times for 10 min each. The strains were grown in YMA liquid nutrient medium at 30 °C and 110 rpm for 24–48 h. For inoculation, a 50 mL bacterial suspension with a concentration of 1 × 10^8^ cells/mL was used. Sterile soybean seeds (25 seeds for each variant) were inoculated by immersion in the strain suspension for 2 h. For the control, sterile seeds were soaked in an equal volume of sterile water. Seeds were planted in a 500 mL container with sand in five replicates. Sand was pre-sterilized by heating in a dry-heat oven at 250 °C for 12 h. The plants were grown at a temperature of 22–24 °C during the day and 18–20 °C at night, with a relative humidity of 60–70% and a daylight duration of 14–16 h. Lighting was maintained at a level consistent with the biological requirements of the crop. Watering was provided as needed, maintaining substrate moisture at 60–70% of its full capacity. Watering was carried out with distilled water, and fertilizing was carried out on the 15th and 25th days of cultivation with nitrogen-free Knop solution containing (g/L): K_2_HPO_4_—0.25; MgSO_4_ × 7H_2_O—1; KCl—0.125; FeSO_4_ × 7H_2_O—0.0125. For 30-day-old plants, an analysis of morphometric characteristics and formed nodules was carried out. The obtained data were compared based on the analysis of variance and Duncan’s test (*p* ˂ 0.05) in the Statistica v10 program.

Only seven strains were selected for further analysis. Among the selected strains were those with clear growth-promoting properties, as well as those that had a negative impact on plants. The strains were deposited in the Collection of Rhizosphere Microorganisms of the Institute of Biochemistry and Physiology of Plants and Microorganisms, Russian Academy of Sciences (IBPPM RAS) (Table 1) [31].

### 2.2. Microbiological Characterization

Cultural and morphological characteristics were determined on solid and liquid YMA media. Carbon source utilization was assessed on a minimal agar medium (g/L): tryptone, 1.0; yeast extract, 0.1; carbon source (glucose, arabinose, sucrose, rhamnose, lactose, galactose, maltose, as well as sorbitol, malic acid, and citric acid), 10.0; agar, 15.0. Catalase activity was tested on YMA by adding 3% hydrogen peroxide to bacterial cultures. Denitrification ability was evaluated by growing the bacteria in YMA broth supplemented with 0.1% potassium nitrate for 4 days, followed by the addition of 0.1 mL of Griess reagent. Each isolate was characterized for cultural characteristics of bacterial growth in liquid YMA medium under different pH values (pH range 4–10) and NaCl concentrations (0.5% and 1.0%).

### 2.3. Complete Genome Sequencing and Assembly

Genomic DNA was extracted using the EasyPure Genomic DNA Kit (TransGen Biotech, Beijing, China). Complete genome sequencing was performed using two platforms: DNBSEQ-G50 (MGI, Shenzhen, China) and GridION (Oxford Nanopore Technologies, Oxford, UK). Library preparation was carried out using the MGIEasy FS DNA Library Prep Set, DNBSEQ-G50 High-throughput Sequencing Set (PE150), and SQK-RBK114-96 kits, respectively. Basecalling of Oxford Nanopore reads was performed using dorado v0.8.3 (SUP model). The dna_r10.4.1_e8.2_400bps_sup@v4.3.0 model was used for basecalling. Subsequently, filtlong v0.2.1 [32] was used to remove 5% of reads with the lowest mean Qscore and reads shorter than 2000 nucleotides. Read quality control was performed using fastp and fastplong [33,34]. Metrics of the filtered Oxford Nanopore Technology (ONT, Oxford, UK) reads are presented in Table S1.

Oxford Nanopore reads were assembled using Flye v2.9.2 (options: --nano-hq --read-error 0.03) [35]. Minor assembly errors were corrected by mapping the corresponding MGI reads using Polypolish v0.6.0 [36]. Assembly quality was assessed using BUSCO v6.0.0 with the bradyrhizobium_odb12 dataset. Assembly metrics are provided in Table S2.

Replicon visualization was performed using Galaxy [37,38]. Complete genome sequences of the strains were annotated using the open-source software package Prokka v1.15.2 [39] under the Linux operating system (Ubuntu 22.04).

### 2.4. Replicon Classification and Circularization Validation

Replicons were classified as chromosomal or plasmid based on gene content and assembly structure. Chromosomal sequences were identified by the presence of core housekeeping genes and/or rRNA operons (e.g., *dnaA*, *gyrA*/*gyrB*, *rpoB*, *recA*, 16S/23S rRNA, and *parA*/*parB*), which are widely used as markers of primary bacterial chromosomes. Contigs carrying these markers were interpreted as chromosomal segments, even when present as separate fragments due to unresolved repeat junctions. Putative plasmid replicons were identified by the presence of conjugation and type IV secretion system genes (e.g., *virB*/type IV secretion), consistent with established plasmid replication and mobilization systems, in the absence of a complete chromosomal marker set.

Circular topology was validated by mapping raw Oxford Nanopore reads to the final assemblies using minimap2 with the map-ont preset optimized for nanopore data [40]. Resulting alignments were processed using SAMtools [41]. For each contig, mean sequencing coverage was calculated, and the fraction of secondary alignments (SAM flag 256) was determined following the SAM/BAM specification [42]. Circularization was confirmed by detecting junction-spanning reads, defined as long reads aligning within 5 kb of both the start and end coordinates of a contig.

*De novo* assembly of strains I-2 and I-5 was performed using Flye --nano-raw mode with an expected genome size of ~9–10 Mb and an assembly coverage limit (--asm-coverage 40) to prioritize long reads for repeat resolution. Additional assemblies using read subsets ≥ 20 kb were generated to assess the impact of read length on repeat resolution. Assembly graphs were visually inspected using Bandage [43] to identify structural ambiguities and repeat-mediated junctions.

### 2.5. Phylogenetic and Comparative Genomic Analyses

Average nucleotide identity (ANI) was calculated using the OrthoANI algorithm implemented in ANI Calculator (https://www.ezbiocloud.net/tools/ani, accessed on 7 August 2025) [44]. ANI values recalculated using fastANI v1.34 were highly consistent with those obtained via the EZBioCloud ANI calculator, with differences typically below 0.3% for closely related taxa and below 1% for more distant comparisons. These minor differences did not affect taxonomic conclusions. The digital DNA-DNA hybridization (dDDH) was calculated using the online resource Genome-to-Genome Distance Calculator (GGDC) v3.0 [45]. Pairwise comparison matrices were generated using online resources [46]. Genome BLAST distance phylogeny (GBDP) v2.1 trees were constructed using the Type (Strain) Genome Server (TYGS) platform [45]. For single nucleotide polymorphism (SNP) analysis, contigs of the studied strains were aligned against the genome of the *B. japonicum* type strain USDA 6 (accession number ASM28437v1) using Snippy v4.6 [47]. The search and alignment of genes were performed using MEGA 7 [48]. Phylogenetic trees based on SNP data were constructed in SeaView v5.0.4 [49] using the maximum likelihood method with the GTR model. Tree visualization was performed using FigTree v1.4.3 [50], resulting in rooted phylogenetic trees. To account for the potential impact of homologous recombination on phylogenetic inference, recombinant regions were detected and removed using Gubbins [51]. Gubbins iteratively reconstructs a maximum likelihood phylogeny, identifies clusters of elevated SNP density indicative of recombination events, masks recombinant regions, and rebuilds the tree until convergence. Sequences with excessive missing data were excluded using a maximum missing data threshold of 31%. The resulting recombination-filtered alignment of polymorphic sites was used for final phylogenetic reconstruction.

Maximum likelihood phylogenetic inference was performed using IQ-TREE2 [52] under the GTR + G nucleotide substitution model. Branch support was assessed using 1000 ultrafast bootstrap replicates and 1000 SH-like approximate likelihood ratio test (SH-aLRT) replicates [53].

Alignment of the nucleotide sequences of the chromosomes was performed using the Mauve program version 2.4.0 with default settings [54].

## 3. Results

**Figure 1:**
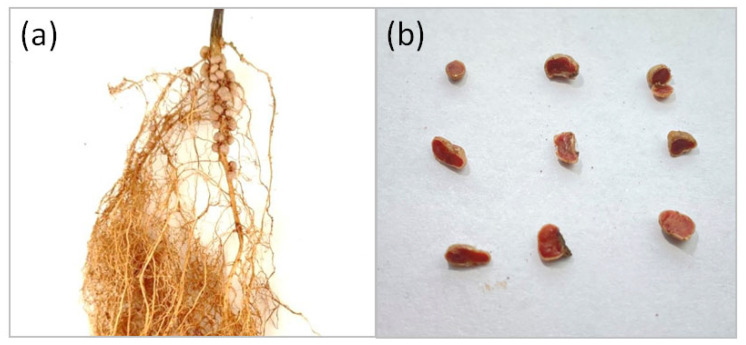
Nodules of soybean plants inoculated with strain II-2. (**a**) Nodules on soybean roots. (**b**) Cross-section of nodules.

To select efficient bacterial strains for use as inoculants, ten pure isolates were obtained from washed and surface-sterilized root nodules of soybean plants (*Glycine max* (L.) Merr.), cultivar Natalie (Figure 1a): I-1, I-2, I-3, I-4, I-5, I-6, II-1, II-2, III-1, and III-2. To select the most promising strains for further analysis, a preliminary assessment of the symbiotic activity of the isolated strains was carried out using the reinoculation method.

### 3.1. Evaluation of Inoculation Effectiveness

Inoculation with strains significantly affected soybean morphometric traits after 30 days of growth (Table 2). The highest dry shoot weight was observed in plants inoculated with strains II-2 (561.6 mg), I-1 (397.6 mg), III-1 (378.4 mg), I-2 (379.0 mg), and II-1 (390.7 mg), all significantly exceeding or equaling the control (283.1 mg). The greatest increase in root weight was recorded for strains I-1 (648.3 mg) and II-2 (488.0 mg), compared with 295.2 mg in the control.

All inoculated variants formed nodules, whereas nodulation was absent in the control. Nodules exhibited an intense red coloration in cross section (Figure 1b), indicating the presence of leghemoglobin in the nodule tissues, which is essential for biological nitrogen fixation.

The highest nodule numbers were detected in plants inoculated with II-2 (25.0 nodules) and III-1 (24.8 nodules). The greatest nodule dry weight was recorded for I-2 (42.9 mg), III-1 (34.6 mg), and II-2 (33.1 mg). Strains III-2 and I-4 demonstrated comparatively low symbiotic efficiency, showing reduced plant weight and lower nodulation parameters.

Overall, strains II-2, III-1, I-2, and I-1 exhibited the strongest positive effects on soybean growth and nodulation. For the next stage of research, not only the strains that demonstrated the greatest effectiveness in promoting plant growth but also those that exerted a growth-inhibiting effect on plants were selected. The inclusion of strains with opposing biological effects in the analysis was necessitated by the need for a comprehensive assessment of the diversity of bacterial associations interacting with soybean plants. This will allow for the examination of growth-promoting and growth-inhibiting effects as manifestations of different strategies of interaction between microorganisms and the host plant.

### 3.2. Phenotypic Characterization of the Strains

**Figure 2:**
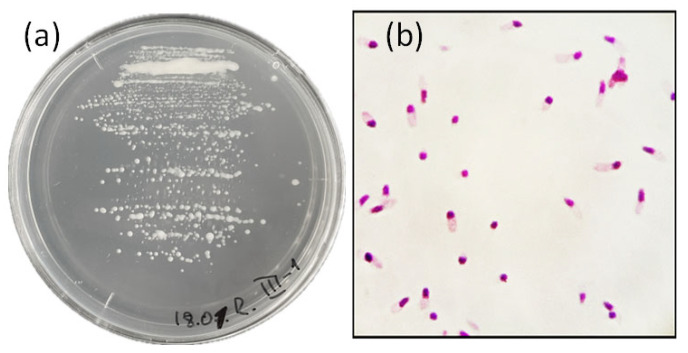
Cultural and morphological features of nodule bacteria on YMA agar medium. (**a**) Bacterial growth on the medium: strain III-1. (**b**) Gram staining.

**Figure 3:**
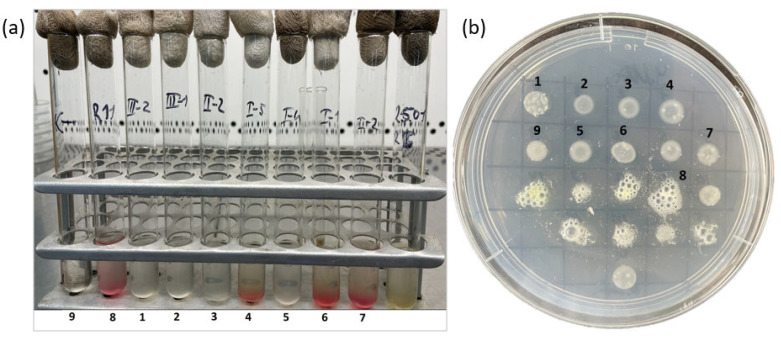
Biochemical characteristics of the strains. (**a**) Evaluation of denitrifying activity. (**b**) Evaluation of catalase activity. 1—strain III-2, 2—III-1, 3—II-2, 4—I-5, 5—I-4, 6—I-1, 7—I-2, 8—positive control, 9—negative control.

Various cultural–morphological and physiological–biochemical characteristics of the selected strains were examined (Table 3). All isolates were slow-growing, with visible colony growth observed after 5–7 days of incubation. The optimal growth conditions for all strains were 28 °C and pH 7.0. All strains were aerobic, Gram-negative rods and exhibited monopolar staining in Gram-stained preparations (Figure 2). Colonies formed by the isolates on solid YMA medium were mucoid, circular, and convex with smooth margins, reaching a diameter of 1–2 mm after 7–9 days of incubation at 28 °C (Figure 2). The strains were able to grow over a pH range of 5–9, forming a homogeneous suspension in liquid YMA medium; at pH 4 and 10, only bottom growth with the formation of flocculent precipitates was observed. Bacterial growth was inhibited at an NaCl concentration of 1% in the medium. The strains actively utilized glucose, arabinose, sucrose, maltose, sorbitol, and galactose as carbon sources; weak growth was observed on rhamnose and lactose, whereas malic and citric acids were not fermented. All strains were catalase-positive, and only three strains (I-1, I-2, and I-5) exhibited denitrifying activity (Figure 3).

### 3.3. Genome Sequencing of the Isolated Strains, Replicon Classification, and Circularization Validation

The genomic characteristics of the isolated strains are summarized in Table 4. Genome sizes showed only minor variation among the strains, ranging from 10.9 Mb (strain III-2) to 11.7 Mb (strain II-2). The number of coding DNA sequences (CDSs) also varied slightly, from 10,807 (strain III-2) to 11,447 (strain II-2). The GC content of all genomes ranged from 63.1% to 63.3%.

Assembly validation metrics are summarized in Table S3. Mean coverage (×) represents the average sequencing depth calculated across each contig after mapping raw ONT reads to the final assembly. The secondary alignment fraction corresponds to the proportion of alignments flagged as secondary (SAM flag 256) and was used as an indicator of repeat-associated or homologous genomic regions. Elevated values suggest structural ambiguity or collapsed repeat sequences. Junction-spanning reads (n) indicate the number of long reads aligning within 5 kb of both the start and end coordinates of a contig. Detection of such reads was considered direct evidence of circular topology.

**Figure 4:**
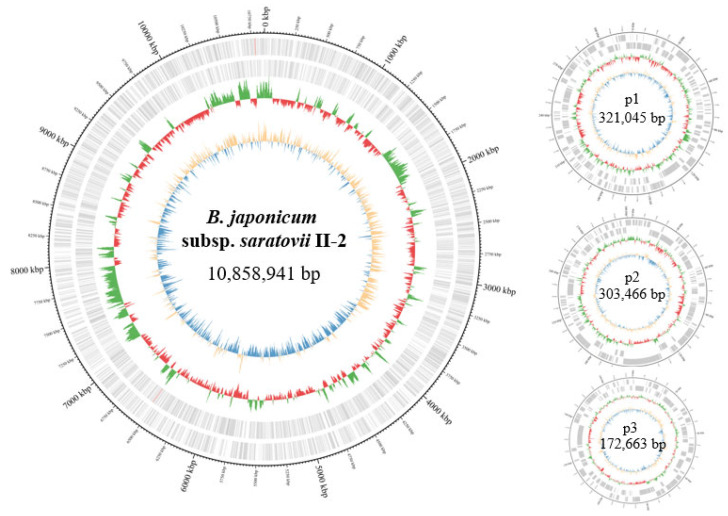
Circular representation of the chromosome and plasmids (strain II-2, NCBI BioProject number PRJNA1258613). The outer ring indicates the genomic coordinates in kilobase pairs. The second ring represents gene density along the chromosome, with coding sequences located on the forward strand shown in green and those on the reverse strand shown in red. The inner ring displays GC skew [(G-C)/(G + C)], where positive values are shown in orange and negative values in blue.

Five strains (I-1, I-4, II-2, III-1, and III-2) yielded fully circularized chromosomes and putative plasmids without structural ambiguities (Figure 4). In contrast, strains I-2 and I-5 each contained a single repeat-mediated chromosomal junction preventing complete circularization. All putative plasmid replicons across all strains were independently validated as circular. The putative plasmids in all strains exhibited GC contents substantially lower than those of the corresponding chromosomes. This observation suggests that these plasmids were likely acquired by the ancestral strains via horizontal gene transfer [57].

Comparison of OrthoANI values among all genomes revealed a high degree of similarity, exceeding 99% (Table 5). However, the alignment fraction (AF) used for genome comparisons was below 70% in most cases. Only the comparisons between strains I-1 vs. I-2 and I-2 vs. I-5 showed AF values above 70% (71.6% and 89.8%, respectively). Alignment of the nucleotide sequences of the chromosomes of the novel strains (Figure S1) did not reveal extended insertions or deletions but showed significant rearrangements of fragments within the chromosomes, which could be reflected in a decrease in AF values. These results indicate that, although the strains are highly similar according to most genomic metrics, the relatively low alignment coverage reflects pronounced genomic individuality among the analyzed strains.

### 3.4. Phylogenetic Analysis Based on SNP Data

**Figure 5:**
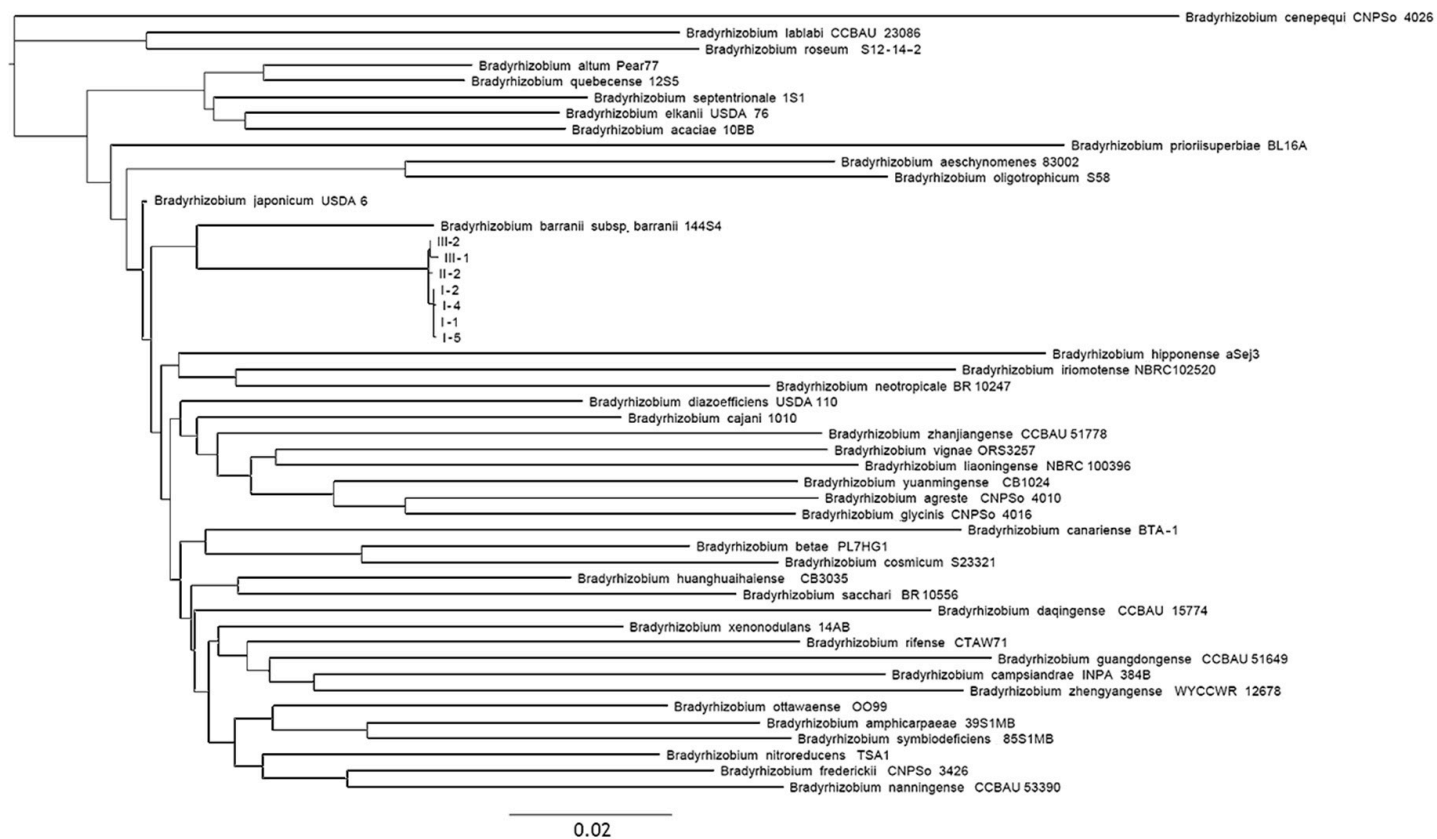
Phylogenetic relationships of the studied strains to the type strains of *Bradyrhizobium* spp. based on whole genome analysis of 5351 identified core SNPs. The dendrogram was constructed using SeaView v5.0.4 with 500 bootstrap support, the maximum likelihood method, and the GTR model. The scale bar indicates 0.02 substitutions per nucleotide position.

To further analyze the phylogenetic affiliation of the seven representative *Bradyrhizobium* sp. isolates, a phylogenetic tree was constructed based on single nucleotide polymorphism (SNP) analysis. For this purpose, reference strains of the genus *Bradyrhizobium* were retrieved from the NCBI international nucleotide sequence database. The SNP-based approach enables genome-wide comparisons of strains based on core single nucleotide polymorphisms. In total, 5351 single nucleotide substitutions were identified. The results of the analysis showed that all of the novel strains clustered into a distinct group (Figure 5), consistent with the results obtained from *rrs* gene analysis, and confirmed their affiliation with the genus *Bradyrhizobium*. However, based on branch lengths in the SNP-based phylogenetic tree, the two closest related species to novel strains were identified as *B. japonicum* and *B. barranii*.

### 3.5. Average Nucleotide Identity (ANI) Analysis

At present, Average Nucleotide Identity (ANI) is considered one of the most important criteria for determining the taxonomic position of microorganisms. The generally accepted threshold value for species boundries based on ANI is 95%. All novel strains and the type strains of species within the genus *Bradyrhizobium* were analyzed using the OrthoANI test (Table 6). The 95% threshold was exceeded in comparisons with the *B. japonicum* type strain USDA 6^T^ and with the type strains of the subspecies *B. barranii* subsp. *barranii* and *B. barranii* subsp. *apii*.

**Figure 6:**
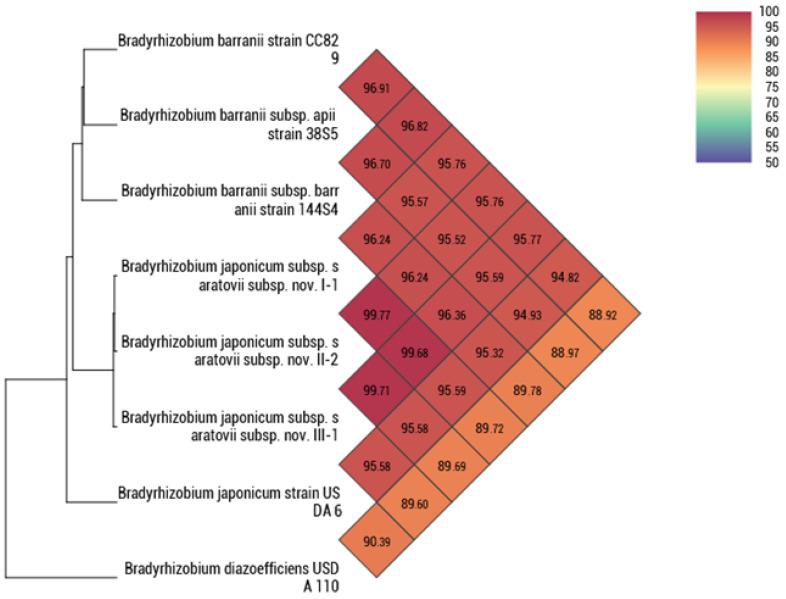
Heat map and dendrogram showing the distribution of strains based on average nucleotide identity values. The genome of *B. diazoefficiens* strain USDA 110 was used as the root.

Figure 6 clearly demonstrates the separation of *Bradyrhizobium* strains into distinct groups, in which representative reference strains with the highest ANI values were selected. For clarity, strains with ANI values > 95% were included: three strains of *B. barranii* (isolated in Canada and Australia) and the *B. japonicum* type strain USDA 6^T^. *B. diazoefficiens* USDA 110^T^ was used as the strain with the highest ANI value among those with ANI ≤ 95%. The positioning of the strains on the dendrogram is consistent with the results obtained using other obtained methods. All *B. barranii* strains form a separate clade in the dendrogram, novel strains occupy an intermediate position between *B. barranii* and *B. japonicum*, and *B. diazoefficiens* USDA 110 is located as the root of the tree.

**Figure 7:**
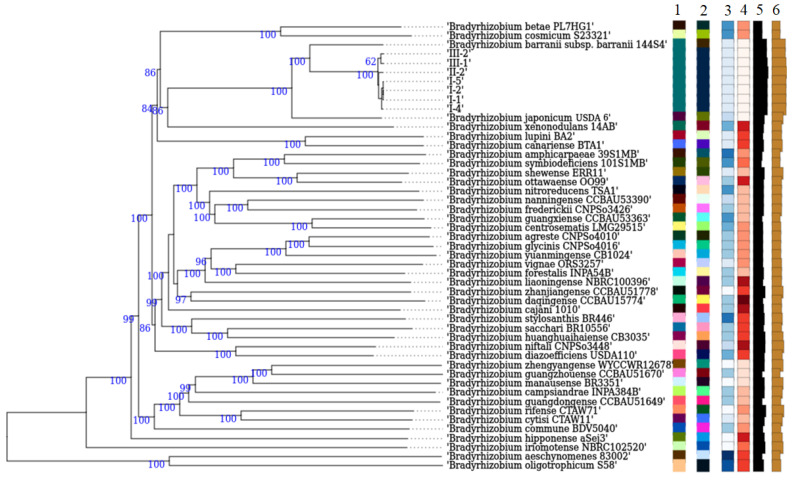
Phylogenetic tree of GBDP intergenic distances based on the whole genome sequences of novel strains and 42 type strains of the genus *Bradyrhizobium*. Numbers above the branches represent pseudo-bootstrap GBDP support values > 60% out of 100 replicates, with an average branch support of 89.9%. Colors from left to right indicate membership in species clusters (1), subspecies clusters (2), G + C percentage (3), delta statistics (4), genome size (in bp, 5), and protein abundance (6).

In agreement with the previous results, the analysis of representative strains using the TYGS platform yielded similar findings (Figure 7). All isolated strains occupy an intermediate phylogenetic position between the two species *B. barranii* and *B. japonicum*. Strains I-1, I-2, I-4, I-5, II-2, III-1, and III-2, together with *B. barranii* 144S4^T^, form a distinct species-level group, within which novel strains are assigned, according to the TYGS results, to a different (novel) subspecies.

Based on comparative genome analyses using ANI, GBDP, and SNP approaches, the strains isolated in this study occupy an intermediate (nearly equidistant) phylogenetic position between the previously described species *B. japonicum* and *B. barranii*, forming a distinct cluster in phylogenetic trees. However, because ANI values between the new strains and the type strains of *B. japonicum* and *B. barranii* exceed 95%, the proposal of a novel species is not justified. At the same time, assignment of the new strains to the species *B. barranii* is also questionable, as this would result in an excessive phylogenetic proximity between the species *B. japonicum* and *B. barranii*.

Given that the ANI value between the type strains of *B. japonicum* and *B. barranii* is 95.2%, a fact that was not considered during the description of *B. barranii* in 2022 [55], despite ANI analysis already being recommended for the description of new bacterial species at that time, we propose that *B. barranii* should be regarded as a later heterotypic synonym of *B. japonicum*. Nevertheless, due to the observed differences between these bacterial groups, we propose to subdivide the species *B. japonicum* into the following subspecies: *Bradyrhizobium japonicum* subsp. *japonicum* subsp. nov., *Bradyrhizobium japonicum* subsp. *barranii* comb. nov., and *Bradyrhizobium japonicum* subsp. *apii* comb. nov. The seven strains isolated in this study, which form a distinct lineage within *Bradyrhizobium japonicum*, are proposed to represent a new subspecies, *Bradyrhizobium japonicum* subsp. *saratovii* subsp. nov.

The genome of strain CC829 is positioned on a separate branch relative to the other strains proposed as subspecies of *B. japonicum*, with ANI values below 97% (Figure 7). This indicates that strain CC829, which is used in commercial inoculants for legumes, belongs to a distinct genomic group within the species *B. japonicum* [58].

### 3.6. Phylogenetic Analysis of B. japonicum Strains

**Figure 8:**
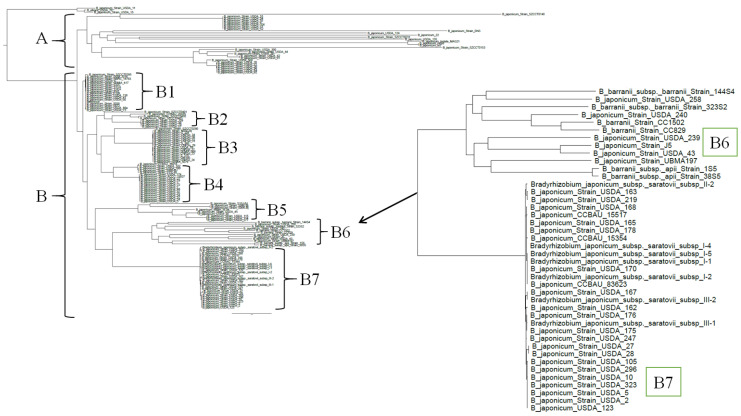
Phylogenetic relationships of *B. japonicum* and *B. barranii* strains based on whole genome analysis of 18,976 identified core SNPs. The maximum likelihood method with the GTR model was used using SeaView 5.0.4, with 500 bootstrap support. Letters indicate phylogenetic groups of strains that have ANI values with B. japonicum USDA 6 < 94-95% (group A) and > 94-95% (group B), respectively. Subgroups B1-B7 indicate different genomic clusters within species B. japonicum. All genomic groups and subgroups are presented in detail in the Supplementary Materials (Figures S2–S5, Table S4). Rooting was performed using the *B. elkanii* strain USDA 76.

**Figure 9:**
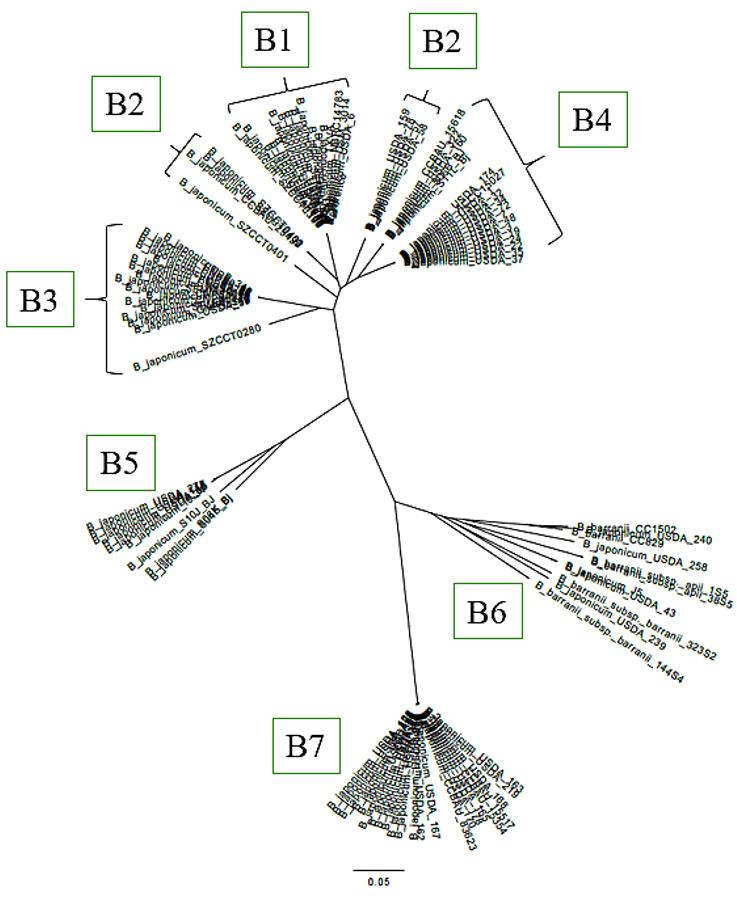
Maximum likelihood phylogeny reconstructed from recombination-filtered core genome SNPs. Recombinant regions were removed using Gubbins, and the tree was inferred in IQ-TREE2 under the GTR + G model with 1000 SH-aLRT and 1000 ultrafast bootstrap replicates. Letters indicate phylogenetic groups of strains that have ANI values with B. japonicum USDA 6 < 94-95% (group A) and > 94-95% (group B), respectively. Subgroups B1-B7 indicate different genomic clusters within species B. japonicum. All genomic groups and subgroups are presented in detail in the Supplementary Materials (Figures S2–S5, Table S4). The scale bar indicates 0.05 substitutions per nucleotide position.

Figure 6, Figure 7 and Figure 8 demonstrate that all of the novel strains occupy an intermediate position between the two species *B. japonicum* and *B. barranii*, as confirmed by ANI analysis. To further clarify the taxonomic placement of the isolated strains, all genomes of *B. japonicum* and *B. barranii* strains available in the NCBI database were compared. As in the previous analyses, a core SNP-based phylogenetic analysis was conducted (Figure 9). The resulting dendrogram clearly illustrates the clustering of strains into phylogenetic lineages. For clarity, the tree was rooted using the genome of the type strain of another species, *Bradyrhizobium elkanii* USDA 76. Nearly all *B. japonicum* strains were divided into two main branches, designated A and B. The *B. japonicum* type strain USDA 6^T^ is positioned in the group B1 at the base of branch B (Figure S2). All seven of the novel strains and twenty-three other strains of *B. japonicum* are located in group B7 (*Bradyrhizobium japonicum* subsp. *saratovii* subsp. nov.) and are confirmed to belong to the species *B. japonicum*, whereas *B. barranii* strains cluster separately from novel strains within a distinct group, B6, together with other *B. japonicum* strains (Figure S3). Branch A contains 29 strains that are clearly separated from all other lineages on the dendrogram (Figure S4). Two strains, *B. japonicum* USDA 15 and *B. japonicum* USDA 11, are located alongside *B. elkanii* USDA 76^T^.

Strains from groups B2, B3, B4, and B5 (Figures S2 and S5) exhibited ANI values above 98% when compared with the *B. japonicum* type strain USDA 6^T^, despite the SNP-based differences observed, confirming their affiliation with the species *B. japonicum*. The results of the ANI analysis are summarized in pairwise comparison matrices (Figure S6). Of the 144 strains analyzed, the majority were identified as *B. japonicum*, including both novel isolates and strains previously classified as *B. barranii*. Thirty-one strains exhibited ANI values below 95% relative to the type strain, consistent with the results of the SNP analysis (Figure 8, Figure 9 and Figures S7 and S8).

Because homologous recombination may bias SNP-based phylogenetic inference in rhizobia, we subsequently performed recombination detection and masking using Gubbins. The recombination-filtered alignment was used to reconstruct a second maximum likelihood tree with branch support assessment (Figure 9). Strains showing ANI values below 95% relative to the *B. japonicum* type strain in the initial SNP tree (branch A) were excluded by Gubbins at the alignment filtering stage due to elevated levels of missing data. The exclusion of these genomes did not alter the phylogenetic placement of the remaining strains. Comparison of the unfiltered and recombination-filtered phylogenies demonstrated overall topological congruence. Our strains formed a distinct and well-supported clade in both trees.

Based on the ANI, dDDH, and SNP test results, the reclassification was proposed for 23 of the 31 analyzed strains of *B. japonicum* (Table 7). Consequently, we propose a reconsideration of the taxonomic status of these 31 strains and their reassignment according to the obtained results. For the remaining eight strains, for which no closely related species could be identified among other *Bradyrhizobium* species, we propose classification as *Bradyrhizobium* sp., without specifying a species-level affiliation.

## 4. Discussion

It is well established that the key bacterial inoculants used for soybean cultivation predominantly belong to the genera *Bradyrhizobium* and *Sinorhizobium* [59,60]. *Bradyrhizobium* represents a highly diverse bacterial genus that includes species of considerable economic importance due to their ability to fix atmospheric nitrogen and to form effective symbiotic associations with leguminous crops used in agriculture [27,29,55]. However, the efficiency of biological nitrogen fixation is closely linked to the selection of highly effective strains, preferably originating from local populations. Therefore, expanding the known diversity of rhizobia through the identification and characterization of naturally occurring strains is essential.

In the present study, ten pure-culture strains of root nodule bacteria were isolated from surface-sterilized nodules of soybean (*Glycine max* (L.) Merr.) cultivar Natalie, grown in a greenhouse using field soil from the Lower Volga region. In the Saratov region, soybean is an introduced crop and thus requires the implementation of effective cultivation strategies adapted to local conditions. At this time, no data have been reported in the scientific literature regarding the isolation of rhizobial strains from the Lower Volga region, highlighting the novelty and regional relevance of the present study.

At the initial stage of the study, the symbiotic activity of ten isolated strains was evaluated using a reinoculation assay on soybean plants. Based on the morphometric parameters of inoculated plants, seven strains were selected for further analysis. The selection included strains that promoted plant growth as well as those showing neutral or negative effects. The inclusion of strains with contrasting symbiotic performance was considered important for a comprehensive analysis of plant–microbe interactions and for identifying factors determining effective or ineffective symbiosis. The observed variation in plant weight and nodulation parameters indicates substantial differences in symbiotic efficiency among the tested strains.

Strains II-2 and III-1 combined high nodule number with increased plant weight, suggesting effective nitrogen fixation and well-established symbiotic compatibility. Notably, strain I-2 formed fewer nodules than II-2 but produced the highest nodule dry mass, which may indicate larger or more physiologically active nodules. The high root weight observed for strain I-1 may reflect enhanced root system development associated with effective rhizobial signaling and early symbiotic establishment. In contrast, strains III-2 and I-4 showed limited promotion of plant growth and lower nodulation intensity, suggesting reduced symbiotic performance under the experimental conditions. Overall, the results demonstrate that symbiotic effectiveness among the studied strains is strongly strain-dependent and highlight the importance of selecting highly efficient strains for further functional and genomic analyses. Although long-read sequencing enabled near-complete assemblies for all strains, strains I-2 and I-5 each contained a single unresolved chromosomal junction associated with repetitive genomic regions.

In strain I-2, the unresolved region corresponded to an ~87 kb sequence exhibiting approximately twofold higher coverage relative to the chromosomal average. This coverage pattern, together with the assembly graph structure, indicates the collapse of a near-identical chromosomal duplication. Only one assembled copy of this repeat was present in the final assembly, preventing unambiguous circularization of the chromosome. In strain I-5, the chromosomal ambiguity was caused by two large homologous regions (~80–90 kb) forming a bifurcation in the assembly graph. Unlike strain I-2, no coverage doubling was observed, suggesting the presence of diverged but highly homologous segments rather than identical duplicated copies. Such repeat-mediated junctions are known limitations even in long-read assemblies when repeat length approaches or exceeds effective read length distribution. Importantly, plasmid replicons in both strains were fully circularized and independently validated by junction-spanning long reads. Gene content analysis confirmed the chromosomal identity of the primary replicons. Therefore, the unresolved structures represent repeat-mediated chromosomal configurations rather than additional replicons. Taken together, these findings demonstrate that five genomes are complete and fully circularized, whereas two represent high-quality near-complete assemblies with a single unresolved repeat-mediated chromosomal junction.

Previous studies on *Bradyrhizobium* have demonstrated that the high level of conservation of the 16S rRNA gene does not allow reliable discrimination of strains at the species level [61,62,63,64]. In contrast, SNP-based analyses provide high resolution for both intra- and interspecific differentiation, as well as for phylogenetic inference in bacterial taxa [65]. For example, researchers in Brazil have applied SNP analysis using the Snippy pipeline to detect genetic differences within closely related groups of *Bradyrhizobium* strains, while other researchers have employed SNP-based approaches to identify genetic variation and potential associations with symbiotic phenotypes among *Bradyrhizobium* strains [66,67].

To determine the species and phylogenetic relationships of the *Bradyrhizobium* strains, whole genome data for the same strains were retrieved from the NCBI GenBank database. Based on 5351 identified core SNPs, a non-ultrametric phylogenetic tree was constructed using the maximum likelihood method with the GTR model (Figure 5). On the resulting dendrogram, each reference strain, with the exception of novel isolates, was represented by an individual branch. All strains investigated in the present study formed a tightly clustered group located between the clades corresponding to *B. japonicum* and *B. barranii*. Since each species was represented by a distinct branch on the dendrogram, the intermediate position of novel strains suggests that they either belong to a novel species within the genus *Bradyrhizobium* or represent a transitional group between these two species.

Analysis of average nucleotide identity (ANI) based on whole genome comparisons enables the assessment of genetic and evolutionary distances between strains and provides species and subspecies boundaries, with a widely accepted threshold of 95–96% and 98%, respectively [62,68,69,70,71]. The application of ANI analysis to *Bradyrhizobium* strains has proven effective for identifying novel taxonomic units, particularly among isolates obtained from root nodules of various leguminous plants or from soils. For example, researchers in Morocco employed ANI analysis to compare genomes of indigenous *Bradyrhizobium* species isolated from nodules of *Retama dasycarpa* (L.) Boiss., whereas studies in Brazil applied this approach to investigate *Bradyrhizobium* strains associated with two leguminous hosts, *Vigna unguiculata* (L.) Walp. and *Glycine max* (L.) Merr. [72,73].

Taxonomic identification of the novel strains was further assessed by comparing them with genomes of the type strains retrieved from NCBI. As SNP analysis clustered novel strains together with representatives of both *B. japonicum* and *B. barranii*, the dataset was expanded to include strains belonging to these two species. The results of ANI calculations were consistent with the SNP-based phylogenetic analysis and are summarized in Table 6. Among all analyzed strains, the 95% ANI threshold was exceeded for all strains of *B. japonicum* and *B. barranii*. Given that ANI is widely used for species boundaries and that the highest nucleotide identity values relative to novel strains were observed for *B. barranii* strains, an initial assignment of novel isolates to this species could be considered. However, when a reduced set of strains with ANI values above 95% was analyzed, all the novel strains consistently occupied an intermediate position between *B. japonicum* and *B. barranii* on the phylogenetic dendrograms (Figure 6 and Figure 7).

To corroborate the results obtained from SNP-based phylogeny and ANI analyses, a genome-scale phylogenetic tree was constructed using the genome BLAST distance phylogeny (GBDP) approach implemented in the TYGS platform. According to the results, all isolated strains occupy an intermediate position between the two species *B. barranii* and *B. japonicum* (Figure 7). However, the TYGS analysis revealed a highly supported clade corresponding to the species cluster representing *B. barranii*, which included all seven of the novel strains. Within this clade, strains I-1, I-2, I-4, I-5, II-2, III-1, and III-2 formed a distinct sub-branch separated from the type strain *B. barranii* 144S4. According to TYGS, this sub-branch corresponds to a different (novel) subspecies.

Because novel strains consistently occupied an intermediate phylogenetic position between *B. japonicum* and *B. barranii*, pairwise comparisons were performed for all available strains of these two species together with novel strains. For this purpose, 130 genomes of *B. japonicum* and six genomes of *B. barranii* were retrieved from the NCBI database. A non-ultrametric phylogenetic tree was constructed using a maximum likelihood SNP-based approach based on 18,976 identified core SNPs (Figure 8). The genome of *B. elkanii* USDA 76 was used as the outgroup to root the tree.

SNP-based analysis enables the detection and alignment of shared core SNPs across large strain datasets and represents a promising approach for microbial differentiation and phylogenetic inference [65,74,75]. The resulting dendrogram clearly demonstrates the separation of strains into distinct phylogenetic lineages. If novel strains belonged to a novel species, they would be expected to cluster near the root of the tree alongside *B. elkanii* USDA 76 or to occupy a position intermediate between the root and *B. japonicum* strains. However, all of the novel strains are deeply nested within the tree, forming group B7 among other *B. japonicum* strains. Similarly, all six *B. barranii* strains are also located within the tree, clustering in the group B6 together with additional *B. japonicum* strains.

The branching pattern of the dendrogram reflects a hierarchical structure based on shared SNPs, with more closely related strains forming tighter clusters. The *B. japonicum* type strain USDA 6^T^ is positioned at the base of branch B within group B1. Based on the overall topology of the tree, it can be inferred that all strains located below group B1, corresponding to the entire B branch, belong to the species *B. japonicum*, including both novel strains and all strains previously classified as *B. barranii*. Accordingly, *B. barranii* should be regarded as a later heterotypic synonym of *B. japonicum*. In addition, 29 strains assigned to *B. japonicum* formed a separate branch (A). If this interpretation is accepted, strains belonging to branch A do not, in fact, represent *B. japonicum*. Two other strains, *B. japonicum* USDA 15 and *B. japonicum* USDA 11, occupied a basal position together with *B. elkanii* USDA 76 at the root of the tree. Overall, these results suggest that not all strains currently assigned to *B. japonicum* genuinely belong to this species and that a comprehensive taxonomic revision is required. The same conclusion applies to strains currently classified as *B. barranii*.

Because homologous recombination can influence SNP-based phylogenetic inference, particularly in rhizobia, recombinant regions were subsequently identified and removed using Gubbins prior to final tree reconstruction. This approach reduces the impact of horizontally acquired genomic segments and allows phylogenetic relationships to be inferred primarily from vertically inherited variation.

Importantly, the overall topology of the phylogeny remained consistent after recombination filtering (Figure 8 and Figure 9). The newly sequenced strains continued to form a distinct and well-supported monophyletic group, indicating that their phylogenetic placement is not attributable to recombinant regions. This topological stability supports the robustness of the observed clustering pattern.

Genomes comprising branch A in the initial tree (Figure 8)—characterized by lower ANI values relative to the type strain—were excluded by Gubbins during the alignment filtering stage due to elevated levels of missing data (>48%). The exclusion of these genomes did not alter the structure of the remaining phylogeny or the position of the focal clade (Figure 9). Their removal is therefore interpreted as a consequence of alignment incompleteness rather than as a biologically driven reassignment. Overall, the concordance between ANI analysis and recombination-filtered SNP phylogeny supports the presence of a genomically coherent lineage among the newly sequenced strains.

Taxonomic identification of strains is mainly performed by whole-genome analysis using the ANI and GGDC tests, for which numerical values for species boundaries are available [24,62,71,76]. However, for strains with which the ANI and GGDC values that lie right on the species boundary, there are difficulties in assigning them to one or another species. Such difficulties were also encountered in our work. The novel isolated strains, based on the ANI and GGDC test results, can be assigned to either species: *B. barranii* or *B. japonicum*. We carefully examined the arguments in the work of Bromfield et al. (2022) [55] regarding the description of the *B. barranii* species. ANI values for B. barranii strains with *B. japonicum* type strain USDA 6^T^ can be assessed ambiguously: both for strains of a new species (ANI values < 95–96% [69]) and for strains of the same species (ANI values > 94.5% [24,26]). The results of digital DDH are also ambiguous. On the one hand, the DDH value is 63.60% [60.7–66.4%], which is below 70% for the species boundary. On the other hand, the probability that DDH > 70% (i.e., same species) is 63.6% (via logistic regression). Furthermore, phylogenetic trees constructed based on the analysis of the genomes of all strains of the *B. barranii* and *B. japonicum* species available in GenBank (Figure 8) show the absence of distinctness of *B. barranii* strains from *B. japonicum* strains. Assigning strains of clade B7 to either the *B. barranii* or *B. japonicum* species would, in our opinion, hinder the use of ANI and GGDC tests for the taxonomic identification of strains of this group of bacteria. Thus, based on bioinformatical analysis, we suggest that *B. barranii* is a later heterotypic synonym of *B. japonicum*, which should take precedence. ANI value analysis supports the reassignment of *B. barranii* subsp. *barranii* and *B. barranii* subsp. *apii* to *B. japonicum* subsp. *barranii* and *B. japonicum* subsp. *apii*, respectively.

We propose to assign all strains of *B. japonicum* that have an ANI value with *B. japonicum* type strain USDA 6^T^ of more than 98% to *B. japonicum* subsp. *japonicum*. We propose to name clade B7, containing 23 strains of *B. japonicum* and novel strains isolated from soybean nodules in the Lower Volga region, as *B. japonicum* subsp. *saratovii* subsp. nov. in honor of the Saratov region.

Based on the core SNP analysis, it can be inferred that 31 strains currently named as “*B. japonicum*” but not clustering within branch B likely belong to other species. To test this hypothesis, pairwise ANI comparisons were performed for all 31 strains. The ANI results were fully consistent with the SNP-based phylogeny. All strains forming the separate A branch (Figure S4) exhibited ANI values below 95% when compared with the *B. japonicum* type strain USDA 6^T^, supporting their exclusion from *B. japonicum* sensu stricto.

For all 31 strains, a comparative analysis of ANI, dDDH, and core SNPs was performed against type strains of other species of the genus *Bradyrhizobium* (Table 7; Figures S7 and S8). Based on the obtained results, a revised taxonomic assignment was proposed for 23 strains previously classified as *B. japonicum*. The strain *B. japonicum* USDA 135 clustered together with the type strain *B. liaoningense* NBRC 100396 on the phylogenetic dendrogram and showed a 100% ANI value, allowing its reassignment to the species *B. liaoningense*. The strain *B. japonicum* USDA 124 clustered with *B. huanghuaihaiense* CB3035 and exhibited an ANI value of 98% relative to this strain, indicating its affiliation with the species *B. huanghuaihaiense*. Seven *B. japonicum* strains (USDA 4, USDA 51, USDA 52, USDA 53, USDA 54, USDA 55, and USDA 106) clustered together with the *B. ottawaense* type strain OO99 and demonstrated ANI values of approximately 99%, supporting their reassignment to the species *B. ottawaense*. Twelve *B. japonicum* strains (USDA 20, USDA 21, USDA 30, USDA 36, USDA 44, USDA 62, USDA 64, USDA 91, USDA 92, USDA 96, USDA 300, and USDA 500) formed a single phylogenetic clade with the *B. diazoefficiens* type strain USDA 110 and showed ANI values ≥ 99%, confirming their assignment to the species *B. diazoefficiens*. Two strains, USDA 15 and USDA 11, clustered with *B. elkanii* USDA 76 and exhibited ANI values ≥ 99%, indicating that they belong to the species *B. elkanii*. For eight strains (22, in8p8, is5, MAG21, SZCCT0148, SZCCT0153, DN3, and SZCCT0231), no type strains of other *Bradyrhizobium* species with ANI values ≥ 95% were identified. Moreover, these strains formed distinct, isolated branches on the phylogenetic dendrogram, separate from other type strains of the genus. Therefore, these strains are recommended to be renamed as *Bradyrhizobium* sp. without assignment to a specific species.

### 4.1. Description of Bradyrhizobium japonicum subsp. japonicum subsp. nov.

*B. japonicum* subsp. *japonicum* (ja.po’ni.cum. N.L. neut. adj. *japonicum*, pertaining to Japan).

The species description is unchanged from its description as *Bradyrhizobium japonicum* [56]. The type strain is strain ATCC 10324^T^ (=CCUG 27876^T^=CIP 106093^T^=DSM 30131^T^=HAMBI 2314^T^=IFO 14783^T^=JCM 20679^T^=LMG 6138^T^=NBRC 14783^T^=NRRL B-4507^T^=NRRL L-241^T^=USDA 6^T^=VKM B-1967^T^), isolated from soybean nodules in Japan.

The GenBank accessions for the complete genome sequence of *B. japonicum* subsp. *japonicum* are PRJDA67463, SAMD00060992, ASM28437v1, and AP012206.

### 4.2. Description of Bradyrhizobium japonicum subsp. barranii comb. nov.

*B. japonicum* subsp. *barranii* (bar.ra’ni.i. N.L. gen. masc. n. *barranii*, of Barran. Named after the late microbiologist Dr. Leslie R. Barran, whose research at Agriculture and Agri-Food Canada led to significant advances in the knowledge of the ecology and genetics of symbiotic nitrogen-fixing bacteria).

The subspecies description is unchanged from its description as *Bradyrhizobium barranii* subsp. *barranii* [55]. The type strain is strain 144S4^T^ (=HAMBI 3722^T^=LMG 31552^T^), isolated from a root nodule of a soybean plant that was inoculated with root-zone soil of *Amphicarpaea bracteata* (L.) Fernald plants growing in Quebec, Canada.

The GenBank accessions for the complete genome sequence of *B. japonicum* subsp. *barranii* are PRJNA714589, SAMN18312838, ASM1756564v3, and CP086136–CP086139.

### 4.3. Description of Bradyrhizobium japonicum subsp. apii comb. nov.

*B. japonicum* subsp. *apii* (a’pi.i. N.L. gen. neut. n. *apii*, of the plant genus *Apios* Fabr.).

The subspecies description is unchanged from its description as *Bradyrhizobium barranii* subsp. *apii* [55]. The type strain is strain 38S5^T^ (=HAMBI 3721^T^=LMG 31556^T^), isolated from a root nodule of a soybean plant that was inoculated with root-zone soil of *Apios americana* Medik. plants growing in Quebec, Canada.

The GenBank accessions for the complete genome sequence of *B. japonicum* subsp. *apii* are PRJNA714594, SAMN18312895, ASM1756568v3, and CP096251–CP096254.

### 4.4. Description of Bradyrhizobium japonicum subsp. saratovii subsp. nov.

*B. japonicum* subsp. *saratovii* (sa.ra.to’vi.i. N.L. gen. neut. n. *saratovii*, from Saratov. Named after the city in which the type strain was isolated).

Strains conform to the species description and exhibit the following characteristics. Strains are slow-growing. On solid YMA medium, colonies appear after 7–9 days at 28 °C, forming sticky, circular, convex colonies with smooth edges, 1–2 mm in diameter. Optimal growth occurs at 28 °C and pH 7. All strains are aerobic, Gram-negative rods, capable of growing in the pH range 4–9. Strains can survive in media containing up to 1% NaCl. Carbon sources utilized include glucose, arabinose, sucrose, maltose, sorbitol, and galactose; in the absence of other carbon sources, strains can also utilize rhamnose and lactose. Malic and citric acids are not used. Strains are catalase-positive; denitrification ability varies among strains. Strains induce nodule formation on the roots of soybean (*Glycine max* (L.) Merr.).

The type strain is strain GmNp2m2^T^ (=II-2^T^=IBPPM 764^T^), isolated from a root nodule of a non-inoculated soybean (*Glycine max* (L.) Merr.) cultivar Natalie that was grown in typical dark chestnut soil in the Saratov region, Russia. The NCBI BioProject accession for the complete genome sequence of *B. japonicum* subsp. *saratovii* is PRJNA1258613.

## 5. Conclusions

As a result of the present study, seven novel strains of root nodule bacteria of the genus *Bradyrhizobium* were isolated and identified from the Lower Volga region, where such strains had not previously been reported. Comparative analyses based on 16S rRNA gene sequences, average nucleotide identity (ANI), genome BLAST distance phylogeny (GBDP), and single nucleotide polymorphism (SNP) analysis demonstrated that the isolated strains occupy an intermediate position between *Bradyrhizobium japonicum* and *Bradyrhizobium barranii*, forming a distinct phylogenetic cluster. At the same time, the obtained genomic data indicate that *B. barranii* is a later heterotypic synonym of *B. japonicum*. Based on the identified genetic differences, subdivision of the species into several subspecies is proposed: *Bradyrhizobium japonicum* subsp. *japonicum* subsp. nov. comb. nov., *Bradyrhizobium japonicum* subsp. *barranii* subsp. nov. comb. nov., *Bradyrhizobium japonicum* subsp. *apii* subsp. nov. comb. nov., and *Bradyrhizobium japonicum* subsp. *saratovii* subsp. nov. The proposed taxonomic revision expands current knowledge of the biodiversity of soybean symbiotic bacteria and contributes to a more accurate understanding of their distribution in previously unexplored regions.

## Figures and Tables

**Table 1:** Strains isolated in this work.

Strain	Collection Acronym	NCBI Acronym	NCBI BioProject Accession
I-1	IBPPM 730	GmNp1m1	PRJNA1253145
I-2	IBPPM 731	GmNp1m2	PRJNA1253146
I-4	IBPPM 732	GmNp1m4	PRJNA1253149
I-5	IBPPM 733	GmNp1m5	PRJNA1253150
II-2	IBPPM 734	GmNp2m2	PRJNA1258613
III-1	IBPPM 735	GmNp3m1	PRJNA1258614
III-2	IBPPM 736	GmNp3m2	PRJNA1258615

**Table 2:** The influence of strains on morphometric traits of soybean plants in a pot experiment.

Variant	Dry Shoot Weight, mg	Dry Root Weight, mg	Number of Tubers, pcs	Dry Weight of Nodules, mg
Control	283.1 ab	295.2 bc	0 a	0 a
III-2	195.8 a	71.2 a	10.40 bc	4.3 ab
I-5	330.5 ab	142.6 ab	11.4 abc	14.7 abc
II-2	561.6 c	488 de	25.0 c	33.1 bc
I-6	350.0 ab	211.9 abc	15.0 abc	16.6 abc
III-1	378.4 abc	326.9 c	24.8 c	34.6 c
I-3	339.4 ab	356.1 cd	18.8 abc	16.6 abc
I-1	397.6 c	648.3 de	17.0 abc	27.7 abc
I-2	379.0 abc	344.1 cd	21.8 bc	42.9 c
I-4	257.0 ab	134.6 ab	5.8 ab	3.7 ab
II-1	390.7 c	137.7 ab	14.2 abc	17.3 abc

Note: variants marked with different letter indices differ significantly according to the results of the analysis of variance (*p* ˂ 0.05).

**Table 3:** Physiological and biochemical characteristics of the studied strains.

Characteristic	Strain								
	I-1	I-2	I-4	I-5	II-2	III-1	III-2	*B. barranii* 144S4^T^ [55]	*B. japonicum* USDA 6^T^ [55,56]
pH 4–9	+	+	+	+	+	+	+	+	+
NaCl (0.5%)	+	+	+	+	+	+	+	+	+
NaCl (1%)	-	-	-	-	-	-	-	-	±
Catalase	+	+	+	+	+	+	+	ND *	ND
Denitrification	+	+	-	+	-	-	-	ND	ND
Glucose	+	+	+	+	+	+	+	-	±
Arabinose	+	+	+	+	+	+	+	ND	+
Galactose	+	+	+	+	+	+	+	-	-
Sucrose	+	+	+	+	+	+	+	-	-
Malic acid	-	-	-	-	-	-	-	-	+
Citric acid	-	-	-	-	-	-	-	-	-
Rhamnose	±	±	±	±	±	±	±	-	-
Lactose	±	±	±	±	±	±	±	-	-
Maltose	+	+	+	+	+	+	+	-	-
Sorbitol	+	+	+	+	+	+	+	+	±

* ND—no data.

**Table 4:** Characteristics of the genomes of the studied strains.

Parameter	I-1	I-2	I-4	I-5	II-2	III-1	III-2
Total length (bp)	11,200,040	11,349,543	11,517,988	11,396,053	11,656,115	11,458,154	10,981,944
Putative plasmids	3	3	4	3	3	3	3
Number of contigs	4	6	5	7	4	4	4
Number of CDS	11,094	11,334	11,337	11,367	11,447	11,371	10,807
Number of tRNAs	61	62	59	62	61	63	57
Number of rRNAs	6	6	6	6	6	6	6
GC content total (%)	63.21	63.22	63.14	63.21	63.13	63.13	63.29
GC content chromosome (%)	63.32	63.57 *	63.35	63.58 *	63.28	63.32	63.40
GC content plasmids (%)	60.4–61.8	60.4–61.8	60.3–61.6	60.4–61.9	60.9–61.2	60.1–61.6	60.9–61.2

* average value of contigs because the chromosome is not complete.

**Table 5:** Comparison of genomes of isolated strains according to the average nucleotide identity and the alignment fraction.

					AF		
Strain	I-1	I-2	I-4	I-5	II-2	III-1	III-2
I-1	-	71.6	68.8	68.7	67.7	63.1	65.6
I-2	99.97	-	66.6	89.8	66.1	62.4	64.5
I-4	99.93	99.94	-	67.1	64.4	63.4	63.4
I-5	99.96	99.98	99.93	-	65.1	62.4	64.7
II-2	99.78	99.80	99.77	99.81	-	65.0	66.9
III-1	99.72	99.73	99.77	99.74	99.74	-	64.8
III-2	99.79	99.70	99.77	99.74	99.70	99.80	-
			**OrthoANI**				

**Table 6:** Average nucleotide identity (OrthoANI, %) values for isolated strains and type strains of closely related species of the genus *Bradyrhizobium*.

Reference Strain	I-1	I-2	I-4	I-5	II-2	III-1	III-2
*B. barranii* ssp. *barranii* 144S4^T^	96.31	96.38	96.32	96.31	96.30	96.36	96.31
*B. barranii* ssp. *apii* 38S5^T^	95.49	95.54	95.53	95.51	95.46	95.54	95.48
*B. japonicum* USDA 6^T^	95.42	95.40	95.36	95.45	95.41	95.43	95.40
*B. diazoefficiens* USDA 110^T^	89.58	89.51	89.49	89.56	89.64	89.47	89.46
*B. ottawaense* OO99^T^	89.02	89.02	89.04	89.08	89.15	89.04	89.08
*B. xenonodulans* 14AB^T^	89.15	89.08	89.04	89.14	89.03	89.05	89.08
*B. niftali* CNPSo 3448^T^	88.74	88.79	88.76	88.77	88.77	88.75	88.78
*B. betae* PL7HG1^T^	88.74	88.76	88.78	88.79	88.76	88.80	88.66
*B. liaoningense* NBRC 100396^T^	88.40	88.42	88.49	88.38	88.58	88.41	88.46
*B. elkanii* USDA 76^T^	80.71	81.16	80.78	80.86	81.33	80.79	81.09

**Table 7:** List of *B. japonicum* strains proposed for the reclassification.

Current Name (Genome Accession Number)	ANI Value with *B. japonicum* USDA 6^T^, %	Proposed Name	dDDH (Identities/HSP Length) Estimate (GLM-Based)	Justification
*B. japonicum* USDA 135 (GCF_000472945.1)	88	*B. liaoningense* USDA 135	99.30% [98.9–99.5%]	ANI value with *B. liaoningense* NBRC 100396 (GCA_030160735.1) = 100%
*B. japonicum* USDA 124 (GCA_000374205.1)	90	*B. huanghuaihaiense* USDA 124	78.10% [75.2–80.8%]	ANI value with *B. huanghuaihaiense* CB3035 (GCA_025200885.1) = 98%
*B. japonicum* USDA 4 (GCA_024170865.1)	90	*B. ottawaense* USDA 4	92.80% [90.8–94.3%]	ANI value with *B. ottawaense* OO99 (GCA_002278135.3) = 99%
*B. japonicum* USDA 51 (GCA_025962335.1)	90	*B. ottawaense* USDA 51	92.80% [90.8–94.3%]	ANI value with *B. ottawaense* OO99 (GCA_002278135.3) = 99%
*B. japonicum* USDA 52 (GCA_025962315.1)	90	*B. ottawaense* USDA 52	92.80% [90.8–94.3%]	ANI value with *B. ottawaense* OO99 (GCA_002278135.3) = 99%
*B. japonicum* USDA 53 (GCA_025962325.1)	90	*B. ottawaense* USDA 53	92.80% [90.8–94.3%]	ANI value with *B. ottawaense* OO99 (GCA_002278135.3) = 99%
*B. japonicum* USDA 54 (GCA_025962375.1)	90	*B. ottawaense* USDA 54	92.80% [90.8–94.3%]	ANI value with *B. ottawaense* OO99 (GCA_002278135.3) = 99%
*B. japonicum* USDA 55 (GCA_025962395.1)	90	*B. ottawaense* USDA 55	92.80% [90.8–94.3%]	ANI value with *B. ottawaense* OO99 (GCA_002278135.3) = 99%
*B. japonicum* USDA 106 (GCA_025961105.1)	90	*B. ottawaense* USDA 106	92.80% [90.8–94.3%]	ANI value with *B. ottawaense* OO99 (GCA_002278135.3) = 99%
*B. japonicum* USDA 20 (GCA_024171125.1)	90	*B. diazoefficiens* USDA 20	100.00% [100–100%]	ANI value with *B. diazoefficiens* USDA 110 (GCA_001642675.1) = 100%
*B. japonicum* USDA 21 (GCA_024171105.1)	90	*B. diazoefficiens* USDA 21	99.90% [99.9–100%]	ANI value with *B. diazoefficiens* USDA 110 (GCA_001642675.1) = 100%
*B. japonicum* USDA 30 (GCA_024171185.1)	90	*B. diazoefficiens* USDA 30	99.80% [99.7–99.9%]	ANI value with *B. diazoefficiens* USDA 110 (GCA_001642675.1) = 100%
*B. japonicum* USDA 36 (GCA_025962255.1)	90	*B. diazoefficiens* USDA 36	89.40% [87.1–91.4%]	ANI value with *B. diazoefficiens* USDA 110 (GCA_001642675.1) = 99%
*B. japonicum* USDA 44 (GCA_025962295.1)	90	*B. diazoefficiens* USDA 44	89.40% [87.1–91.4%]	ANI value with *B. diazoefficiens* USDA 110 (GCA_001642675.1) = 99%
*B. japonicum* USDA 62 (GCA_025962455.1)	90	*B. diazoefficiens* USDA 62	89.00% [86.5–91%]	ANI value with *B. diazoefficiens* USDA 110 (GCA_001642675.1) = 99%
*B. japonicum* USDA 64 (GCA_025962445.1)	90	*B. diazoefficiens* USDA 64	99.80% [99.7–99.9%]	ANI value with *B. diazoefficiens* USDA 110 (GCA_001642675.1) = 100%
*B. japonicum* USDA 91 (GCA_025962555.1)	90	*B. diazoefficiens* USDA 91	88.90% [86.5–91%]	ANI value with *B. diazoefficiens* USDA 110 (GCA_001642675.1) = 99%
*B. japonicum* USDA 92 (GCA_025960915.1)	90	*B. diazoefficiens* USDA 92	99.80% [99.6–99.9%]	ANI value with *B. diazoefficiens* USDA 110 (GCA_001642675.1) = 100%
*B. japonicum* USDA 96 (GCA_025962515.1)	90	*B. diazoefficiens* USDA 96	99.80% [99.6–99.9%]	ANI value with *B. diazoefficiens* USDA 110 (GCA_001642675.1) = 100%
*B. japonicum* USDA 300 (GCA_017831985.1)	91	*B. diazoefficiens* USDA 300	91.10% [88.9–92.9%]	ANI value with *B. diazoefficiens* USDA 110 (GCA_001642675.1) = 99%
*B. japonicum* USDA 500 (GCA_017831945.1)	90	*B. diazoefficiens* USDA 500	97.30% [96.2–98.1%]	ANI value with *B. diazoefficiens* USDA 110 (GCA_001642675.1) = 100%
*B. japonicum* USDA 11 (GCA_024171285.1)	84	*B. elkanii* USDA 11	90.40% [88.2–92.3%]	ANI value with *B. elkanii* USDA 76 (GCA_023278185.1) = 99%
*B. japonicum* USDA 15 (GCA_024170535.1)	84	*B. elkanii* USDA 15	94.60% [92.9–95.9%]	ANI value with *B. elkanii* USDA 76 (GCA_023278185.1) = 99%
*B. japonicum* DN3 (GCA_041357655.1)	88	*Bradyrhizobium* sp. DN3	-	Not found species with value ANI > 95%
*B. japonicum* 22 (GCA_024171145.1)	87	*Bradyrhizobium* sp. 22	-	Not found species with value ANI > 95%
*B. japonicum* in8p8 (GCA_000426845.1)	87	*Bradyrhizobium* sp. in8p8	-	Not found species with value ANI > 95%
*B. japonicum* is5 (GCA_000421305.1)	87	*Bradyrhizobium* sp. is5	-	Not found species with value ANI > 95%
*B. japonicum* MAG21 (GCA_041987565.1)	87	*Bradyrhizobium* sp. MAG21	-	Not found species with value ANI > 95%
*B. japonicum* SZCCT0148 (GCA_018129985.1)	87	*Bradyrhizobium* sp. SZCCT0148	-	Not found species with value ANI > 95%
*B. japonicum* SZCCT0153 (GCA_018129995.1)	89	*Bradyrhizobium* sp. SZCCT0153	-	Not found species with value ANI > 95%
*B. japonicum* SZCCT0231 (GCA_018130245.1)	88	*Bradyrhizobium* sp. SZCCT0231	-	Not found species with value ANI > 95%

## Data Availability

Data are contained within the article and its Supplementary Materials. Additional data supporting the findings of this study are available from the corresponding author upon reasonable request.

## Supplementary Materials

The following supporting information can be downloaded at: https://www.mdpi.com/article/10.3390/microorganisms14030684/s1, Figure S1: Alignment of the nucleotide sequences of the chromosomes of the novel strains I-1, I-2, I-4, I-5, II-2, III-1, and III-2 using the Mauve program; Figure S2: Phylogenetic relationships of *B. japonicum* and *B. barranii* strains based on whole genome analysis of 18,976 identified core SNPs (phylogenetic groups B1, B2, B3); Figure S3: Phylogenetic relationships of *B. japonicum* and *B. barranii* strains based on whole genome analysis of 18,976 identified core SNPs (phylogenetic groups B6 and B7); Figure S4: Phylogenetic relationships of *B. japonicum* and *B. barranii* strains based on whole genome analysis of 18,976 identified core SNPs (phylogenetic group A); Figure S5: Phylogenetic relationships of *B. japonicum* and *B. barranii* strains based on whole genome analysis of 18,976 identified core SNPs (phylogenetic groups B4 and B5); Figure S6: Matrices for comparison of average nucleotide identity of *B. japonicum* strains; Figure S7: Phylogenetic tree of *Bradyrhizobium japonicum* strains based on whole genome analysis of 3559 identified core SNPs; Figure S8: Matrices for comparison of the average nucleotide identity of 31 *B. japonicum* strains together with type strains of other *Bradyrhizobium* species; Table S1: ONT read metrics after fastplong preprocessing; Table S2: Metrics of assembled genomes; Table S3: Validation of genome assembly circularization and repeat structure across seven *Bradyrhizobium* strains; Table S4: Distribution of strains from the GenBank database in genomic groups of the species *Bradyrhizobium japonicum*.

## Abbreviations

The following abbreviations are used in this manuscript:

ANI	Average Nucleotide Identity
dDDH	digital DNA-DNA hybridization
GBDP	Genome BLAST Distance Phylogeny
GGDC	Genome-to-Genome Distance Calculator
ONT	Oxford Nanopore Technology
SNP	Single Nucleotide Polymorphism
TYGS	The Type (Strain) Genome Server
YMA	Yeast Mannitol Agar
