# Supplementary material for: Bacterial Strains from Soybean Nodules in the Lower Volga Region Belong to a New Subspecies Bradyrhizobium japonicum subsp. saratovii subsp. nov
Source: Microorganisms. 2026 Mar 18;14(3):684. doi: 10.3390/microorganisms14030684 (PMC13029459; doi:10.3390/microorganisms14030684)
Supplement: Supplementary file 1 [file microorganisms-14-00684-s001.zip › microorganisms-4171754-supplementary.pdf]

Table S1. ONT read metrics after fastplong preprocessing.

Strain	Bases	Bases with Qscore > 20, %	N50
I-1	1598 million	86	24 thousand
I-2	612 million	86	26 thousand
I-4	430 million	85	29 thousand
I-5	556 million	85	27 thousand
II-2	953 million	85	23 thousand
III-1	1249 million	86	22 thousand
III-2	1001 million	85	26 thousand

Table S2. Metrics of assembled genomes.

Strain	Assembly length	Contigs	N50	Busco-completeness
I-1	11200040	4	10672826	99.1
I-2	11349543	6	9850031	99
I-4	11517988	5	10526943	98.7
I-5	11396053	7	9868638	99.1
II-2	11656115	4	10858941	98.8
III-1	11458154	4	10646748	98.7
III-2	10981944	4	10501890	99.2

Table S3. Validation of genome assembly circularization and repeat structure across seven *Bradyrhizobium* strains. Assembly metrics, read-mapping statistics, and independent circularization validation for all replicons. Circular topology was confirmed by long-read mapping demonstrating reads spanning the circularization junction. Strains I-2 and I-5 contain single unresolved chromosomal repeat-mediated junctions.

Strain	Replicon	Size (bp)	Mean coverage (×)	Secondary alignment fraction	Junction-spanning reads (n)	Structural interpretation	Assembly classification
I-1	Chromosome	10,672,830	143.03	0.047	44	Circular	Complete
	Putative plasmid 1	272,957	137.15	0.064	33	Circular	Complete
	Putative plasmid 2	171,108	121.15	0.123	17	Circular	Complete
	Putative plasmid 3	83,149	42.21	0.142	16	Circular	Complete
I-2	Chromosome	9,849,998	54.01	0.058	0	Repeat-mediated junction	Near-complete
	Putative plasmid 1	273,077	51.44	0.074	16	Circular	Complete (plasmid)
	Putative plasmid 2	171,108	52.06	0.132	3	Circular	Complete (plasmid)
	Putative plasmid 3	83,150	20.81	0.157	3	Circular	Complete (plasmid)
	Repeat node	87,678	16.29	0.769	0	Collapsed duplication	—
I-4	Chromosome	10,526,908	36.69	0.038	9	Circular	Complete
	Putative plasmid 1	323,416	34.37	0.079	7	Circular	Complete
	Putative	275,383	39.52	0.069	10	Circular	Complete

	plasmid 2						
	Putative plasmid 3	222,270	27.32	0.140	4	Circular	Complete
	Putative plasmid 4	169,972	26.92	0.174	7	Circular	Complete
I-5	Chromosome	9,868,471	48.93	0.058	0	Homologous repeat junction	Near-complete
	Putative plasmid 1	273,077	44.14	0.071	9	Circular	Complete (plasmid)
	Putative plasmid 2	166,547	36.48	0.149	7	Circular	Complete (plasmid)
	Putative plasmid 3	83,149	14.28	0.180	6	Circular	Complete (plasmid)
	Repeat node	87,725	14.85	0.757	0	Repeat-mediated	—
II-2	Chromosome	10,858,941	79.91	0.048	17	Circular	Complete
	Putative plasmid 1	321,045	88.36	0.136	19	Circular	Complete
	Putative plasmid 2	303,466	83.65	0.151	34	Circular	Complete
	Putative plasmid 3	172,663	87.65	0.116	8	Circular	Complete
III-1	Chromosome	10,646,748	107.91	0.033	16	Circular	Complete
	Putative plasmid 1	386,385	123.59	0.147	27	Circular	Complete
	Putative plasmid 2	257,513	95.63	0.166	43	Circular	Complete
	Putative plasmid 3	167,508	63.03	0.082	19	Circular	Complete
III-2	Chromosome	10,501,890	89.74	0.037	24	Circular	Complete
	Putative plasmid 1	239,809	105.39	0.074	30	Circular	Complete
	Putative plasmid 2	149,215	78.79	0.146	20	Circular	Complete
	Putative plasmid 3	91,030	35.33	0.107	13	Circular	Complete

Table S4. Distribution of strains from the GenBank database in genomic groups of the species *Bradyrhizobium japonicum*. Strains of groups B1-B5 have ANI values greater than 98% with *B. japonicum* USDA 6^T, respectively, and are assigned to the subspecies *B. japonicum* subsp. *japonicum* subsp. nov. Strains of groups B6 and B7 have ANI values of 94.5-96% with *B. japonicum* USDA 6^T and are assigned to the subspecies *B. japonicum* subsp. *barranii* comb. nov., *B. japonicum* subsp. *apii* comb. nov., and *B. japonicum* subsp. *saratovii* subsp. nov.

**Phylogenetic groups**	**Strains**
**B1** (*B. japonicum* subsp. *japonicum*)	*B. japonicum* SZCCT0395
	B. japonicum DSM 3014
	B. japonicum NBRC 14783
	B. japonicum USDA 6^T
	B. japonicum SEMIA 417
	B. japonicum 532C
	B. japonicum TXVA
	B. japonicum TXEA
	B. japonicum FN1
	B. japonicum E109

	B. japonicum USDA 138
	B. japonicum USDA 41
	B. japonicum USDA 50
	B. japonicum 5038
	B. japonicum 5873
	B. japonicum USDA 66a
	B. japonicum USDA 73
**B2** (*B. japonicum* subsp. *japonicum*)	*B. japonicum* SZCCT0401
	B. japonicum CCBAU 25435
	B. japonicum SZCCT0402
	B. japonicum SZCCT0403
	B. japonicum USDA 159
	B. japonicum USDA 58
	B. japonicum USDA 78
	B. japonicum USDA 57
**B3** (*B. japonicum* subsp. *japonicum*)	*B. japonicum* SZCCT0280
	B. japonicum N03G-Bj
	B. japonicum S 204
	B. japonicum CNPSo 34
	B. japonicum CNPSo 38
	B. japonicum CNPSo 22
	B. japonicum CNPSo 31
	B. japonicum S 340
	B. japonicum CNPSo 29
	B. japonicum USDA 59
	B. japonicum CNPSo 17
	B. japonicum SEMIA 566
	B. japonicum CNPSo 23
	B. japonicum S 370
	B. japonicum GW140
	B. japonicum CNPSo 24
	B. japonicum SEMIA 5079
**B4** (*B. japonicum* subsp. *japonicum*)	*B. japonicum* CCBAU 15618
	B. japonicum USDA 322
	B. japonicum USDA 160
	B. japonicum S06B-BJ
	B. japonicum S11L-Bj
	B. japonicum USDA 174
	B. japonicum ACCC 15027
	B. japonicum USDA 22
	B. japonicum USDA 19
	B. japonicum USDA 1
	B. japonicum USDA 72
	B. japonicum USDA 7
	B. japonicum USDA 48
	B. japonicum USDA 37
	B. japonicum USDA 35
	B. japonicum USDA 34
	B. japonicum USDA 32
	B. japonicum USDA 24
	B. japonicum USDA 3
**B5** (*B. japonicum* subsp. *japonicum*)	*B. japonicum* S10J-BJ
	B. japonicum S04E-Bj
	B. japonicum S06K-Bj
	B. japonicum Is-34
	B. japonicum USDA 45
	B. japonicum USDA 38
	B. japonicum USDA 115
	B. japonicum USDA 114
	B. japonicum USDA 277

**B6** (*B. japonicum* subsp. *barranii*; *B. japonicum* subsp. *apii*; *B. japonicum* CC829)	*B. barranii* subsp. *barranii* 144S4^T
	B. japonicum USDA 258
	B. barranii subsp. *barranii* 323S2
	B. japonicum USDA 240
	B. barranii CC1502
	B. barranii CC829
	B. japonicum USDA 239
	B. japonicum J5
	B. japonicum USDA 43
	B. japonicum UBMA197
	B. barranii subsp. *apii* 1S5
	B. barranii subsp. *apii* 38S5^T
**B7** (*B. japonicum* subsp. *saratovii*)	*B. japonicum* subsp. *saratovii* II-2^T
	B. japonicum USDA 163
	B. japonicum USDA 219
	B. japonicum USDA 168
	B. japonicum CCBAU 15517
	B. japonicum USDA 165
	B. japonicum USDA 178
	B. japonicum CCBAU 15354
	B. japonicum subsp. *saratovii* I-4
	B. japonicum subsp. *saratovii* I-5
	B. japonicum subsp. *saratovii* I-1
	B. japonicum USDA 170
	B. japonicum subsp. *saratovii* I-2
	B. japonicum CCBAU 83623
	B. japonicum USDA 167
	B. japonicum subsp. *saratovii* III-2
	B. japonicum USDA 162
	B. japonicum USDA 176
	B. japonicum subsp. *saratovii* III-1
	B. japonicum USDA 175
	B. japonicum USDA 247
	B. japonicum USDA 27
	B. japonicum USDA 28
	B. japonicum USDA 105
	B. japonicum USDA 296
	B. japonicum USDA 10
	B. japonicum USDA 323
	B. japonicum USDA 5
	B. japonicum USDA 2
	B. japonicum USDA 123

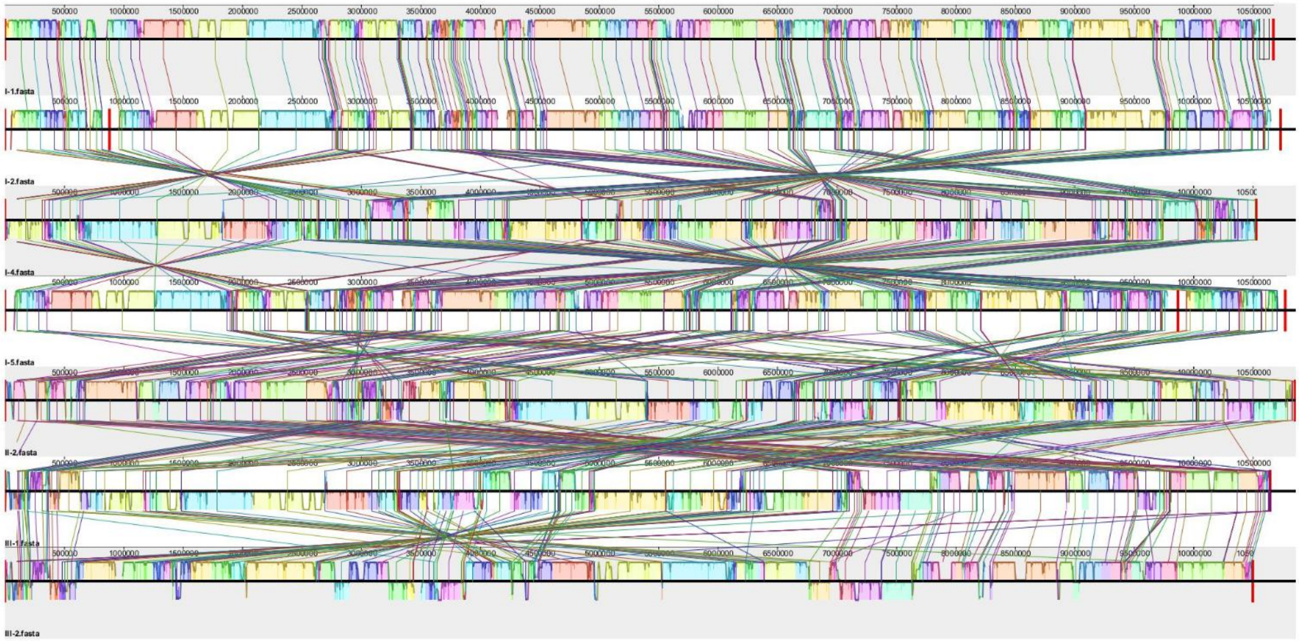

Figure S1. Alignment of the nucleotide sequences of the chromosomes of the novel strains I-1, I-2, I-4, I-5, II-2, III-1, and III-2 using the Mauve program

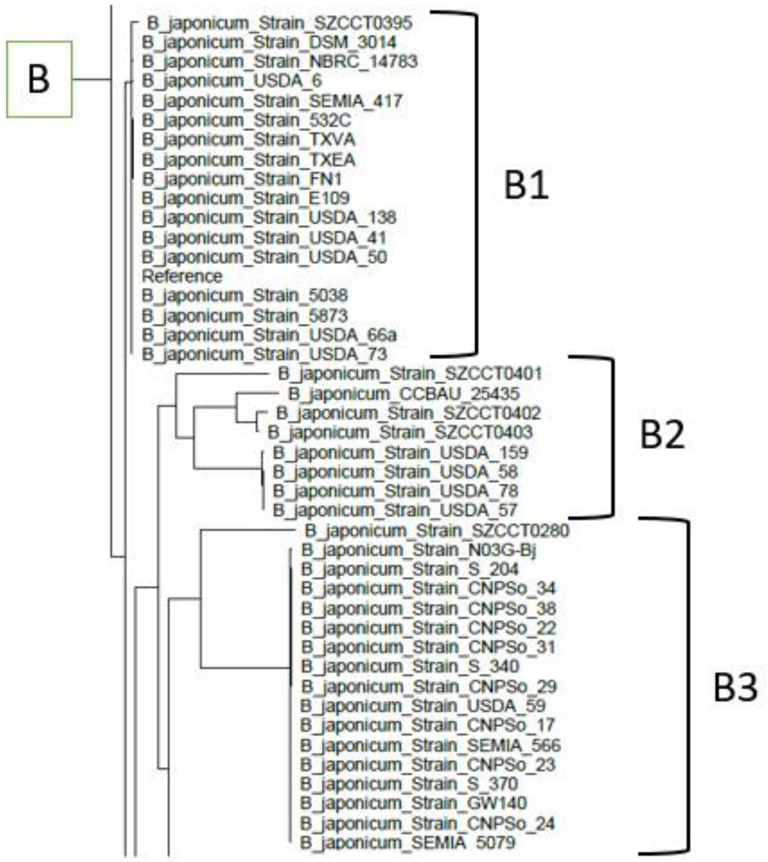

Figure S2. Phylogenetic relationships of *B. japonicum* and *B. barranii* strains based on whole genome analysis of 18,976 identified core SNPs (phylogenetic groups B1, B2, B3).

The maximum-likelihood method with the GTR model was used using SeaView 5.0.4, with 500 bootstrap support. Rooting was performed using *B. elkanii* strain USDA 76.

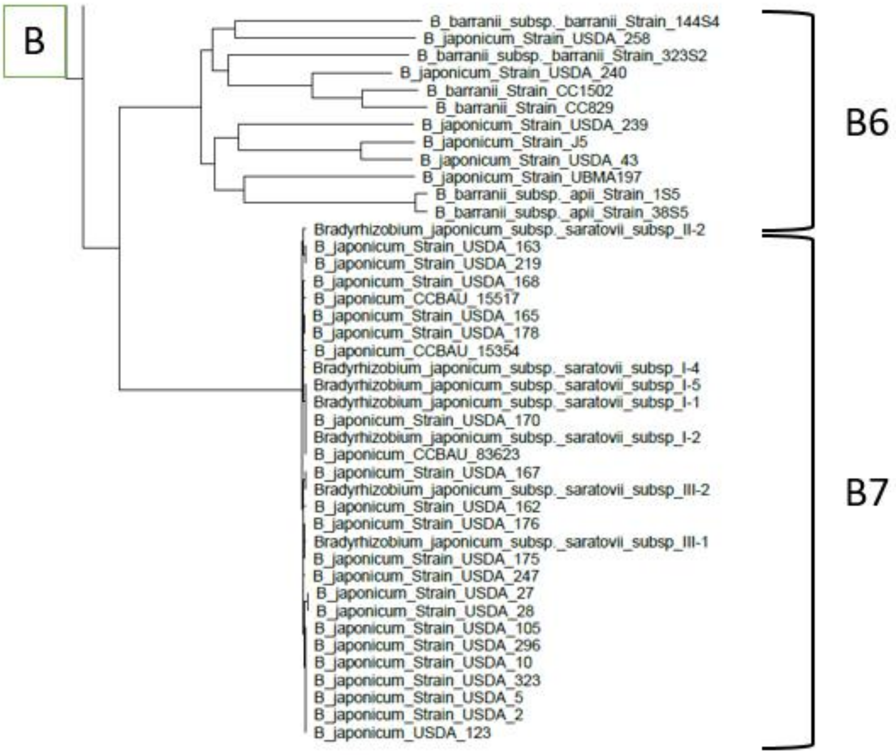

Figure S3. Phylogenetic relationships of *B. japonicum* and *B. barranii* strains based on whole genome analysis of 18,976 identified core SNPs (phylogenetic groups B6 and B7).

The maximum-likelihood method with the GTR model was used using SeaView 5.0.4, with 500 bootstrap support. Rooting was performed using *B. elkanii* strain USDA 76.

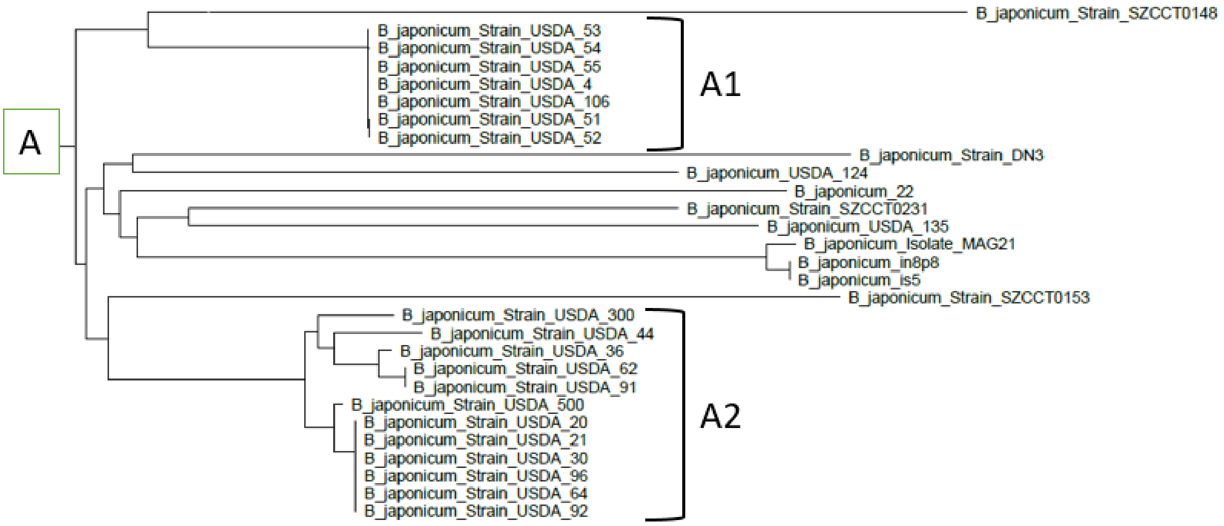

Figure S4. Phylogenetic relationships of *B. japonicum* and *B. barranii* strains based on whole genome analysis of 18,976 identified core SNPs (phylogenetic group A).

The maximum-likelihood method with the GTR model was used using SeaView 5.0.4, with 500 bootstrap support. Rooting was performed using *B. elkanii* strain USDA 76.

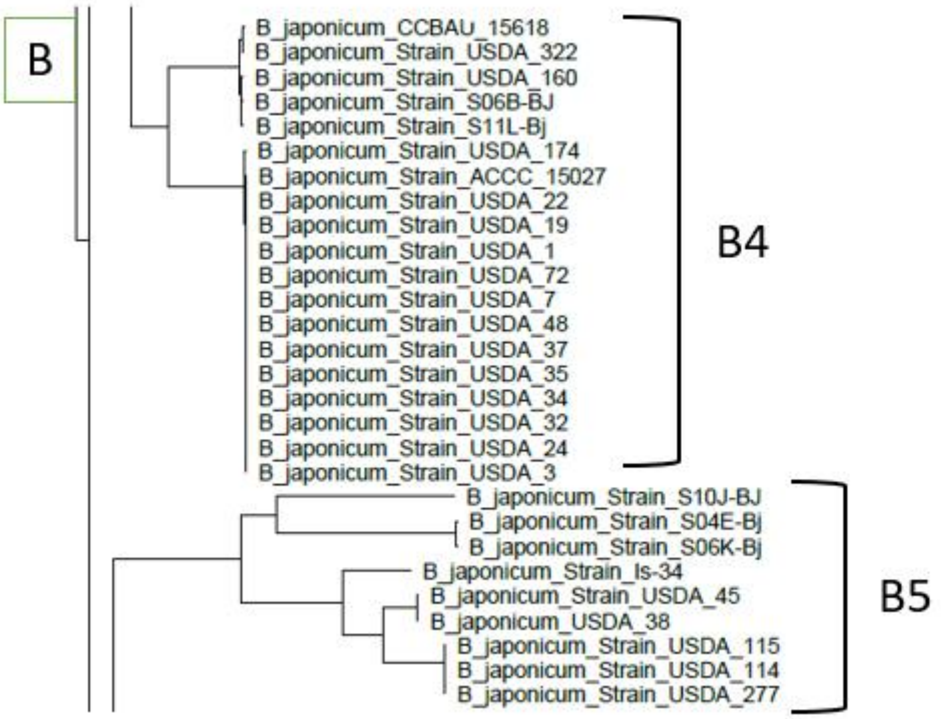

Figure S5. Phylogenetic relationships of *B. japonicum* and *B. barranii* strains based on whole genome analysis of 18,976 identified core SNPs (phylogenetic groups B4 and B5).

The maximum-likelihood method with the GTR model was used using SeaView 5.0.4, with 500 bootstrap support. Rooting was performed using *B. elkanii* strain USDA 76.

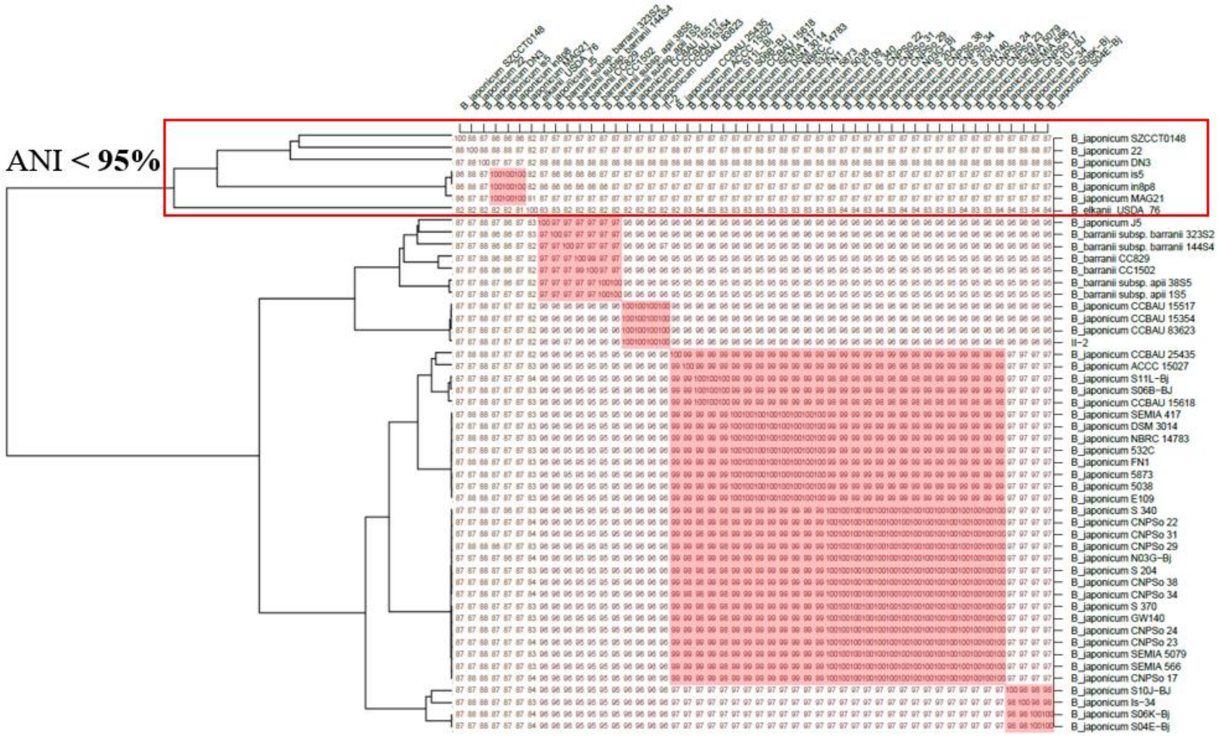

a

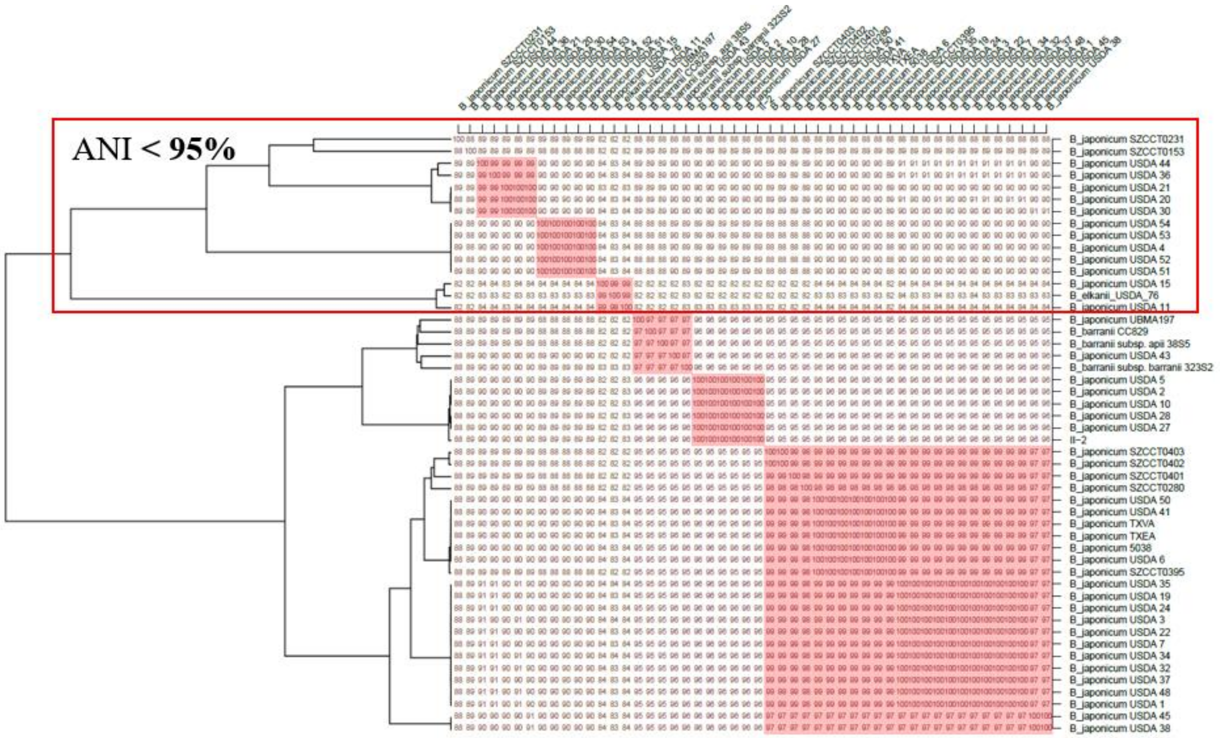

b

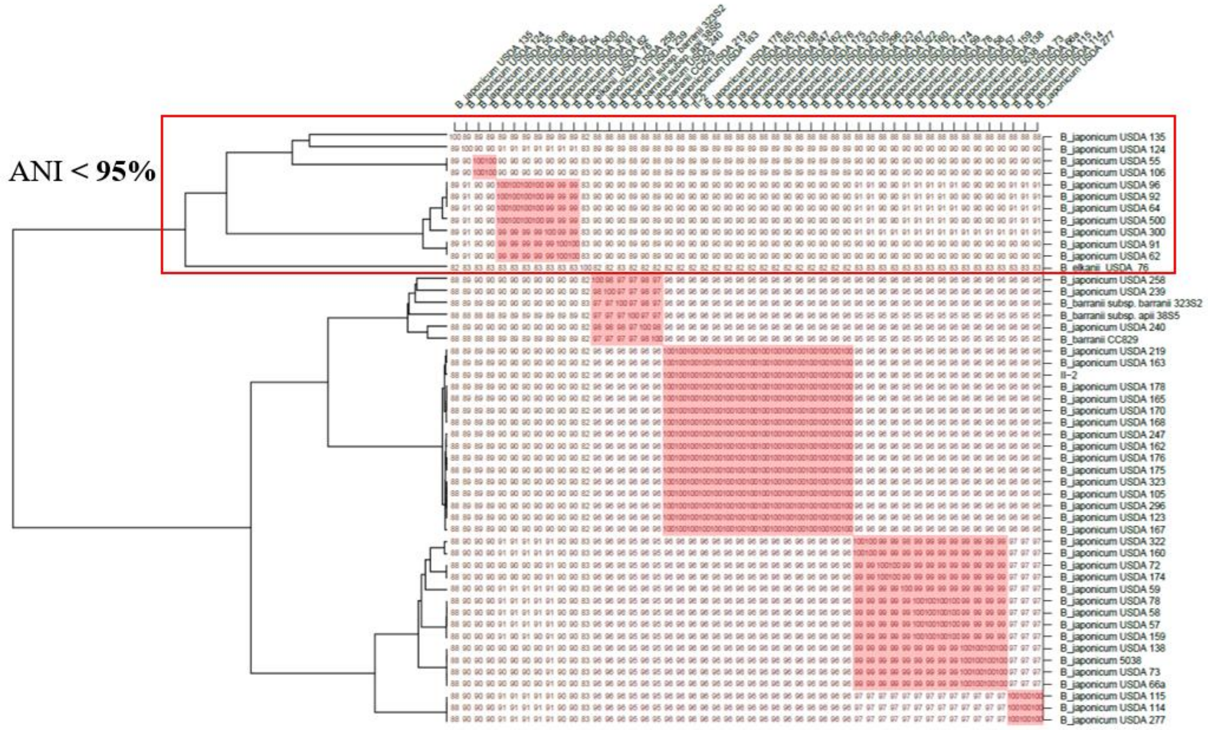

c

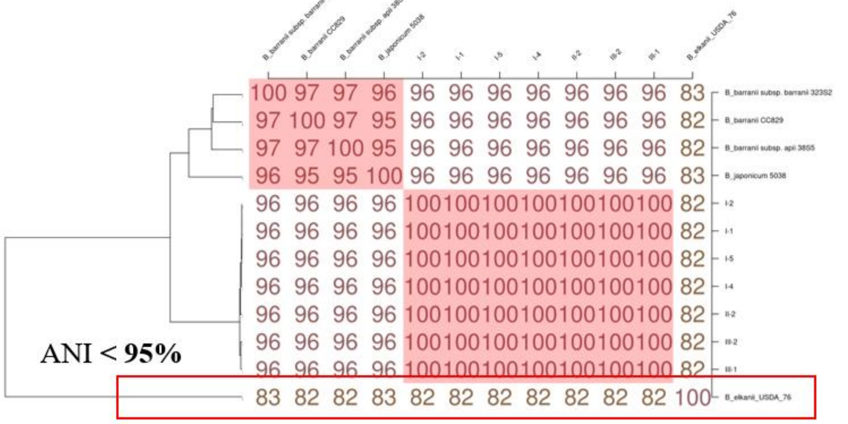

d

Figure S6. Matrices for comparison of average nucleotide identity of *B. japonicum* strains.

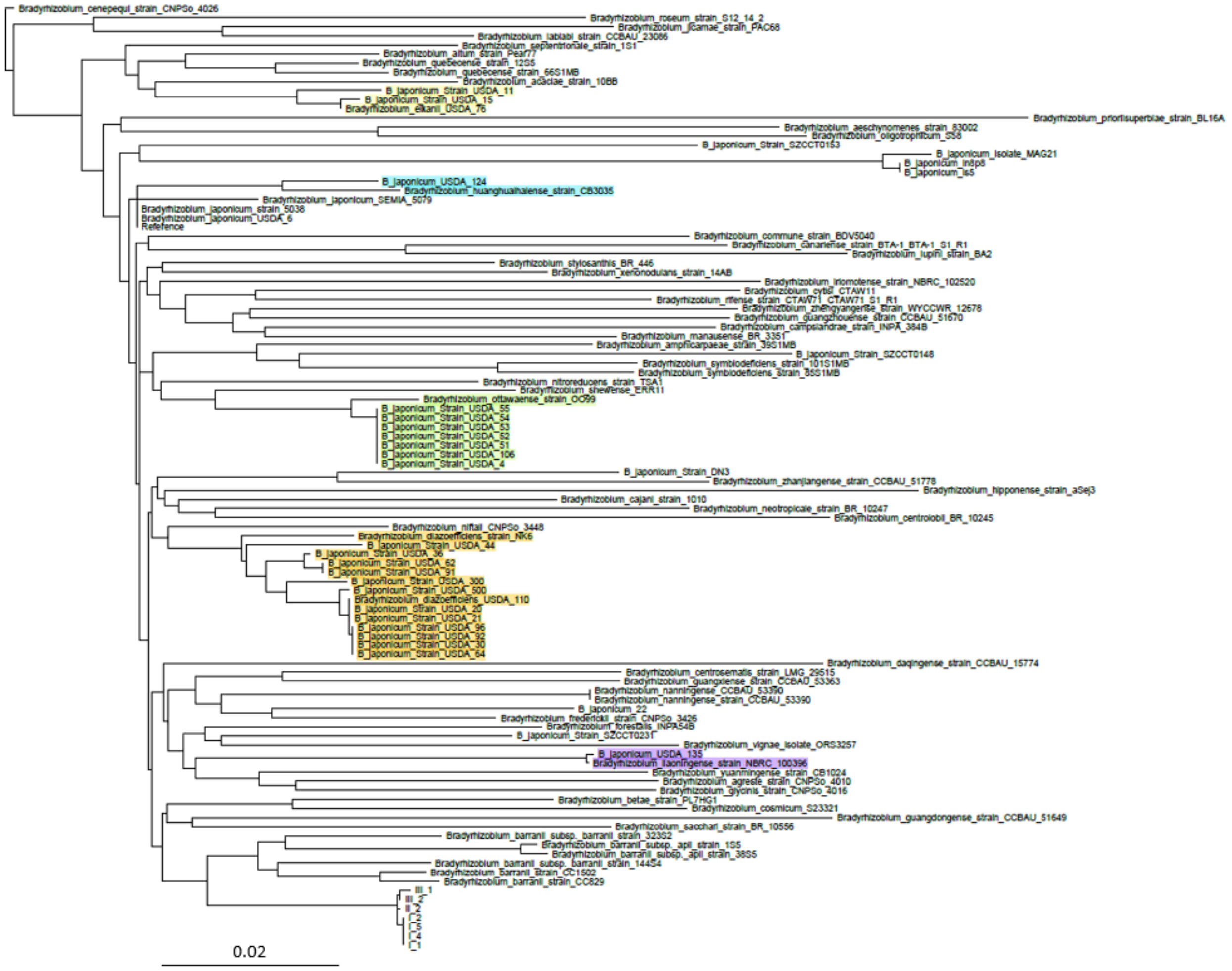

Figure S7. Phylogenetic tree of *Bradyrhizobium japonicum* strains based on whole genome analysis of 3,559 identified core SNPs.

Maximum Likelihood method with the GTR model using SeaView 5.0.4, with 500 bootstrap support.

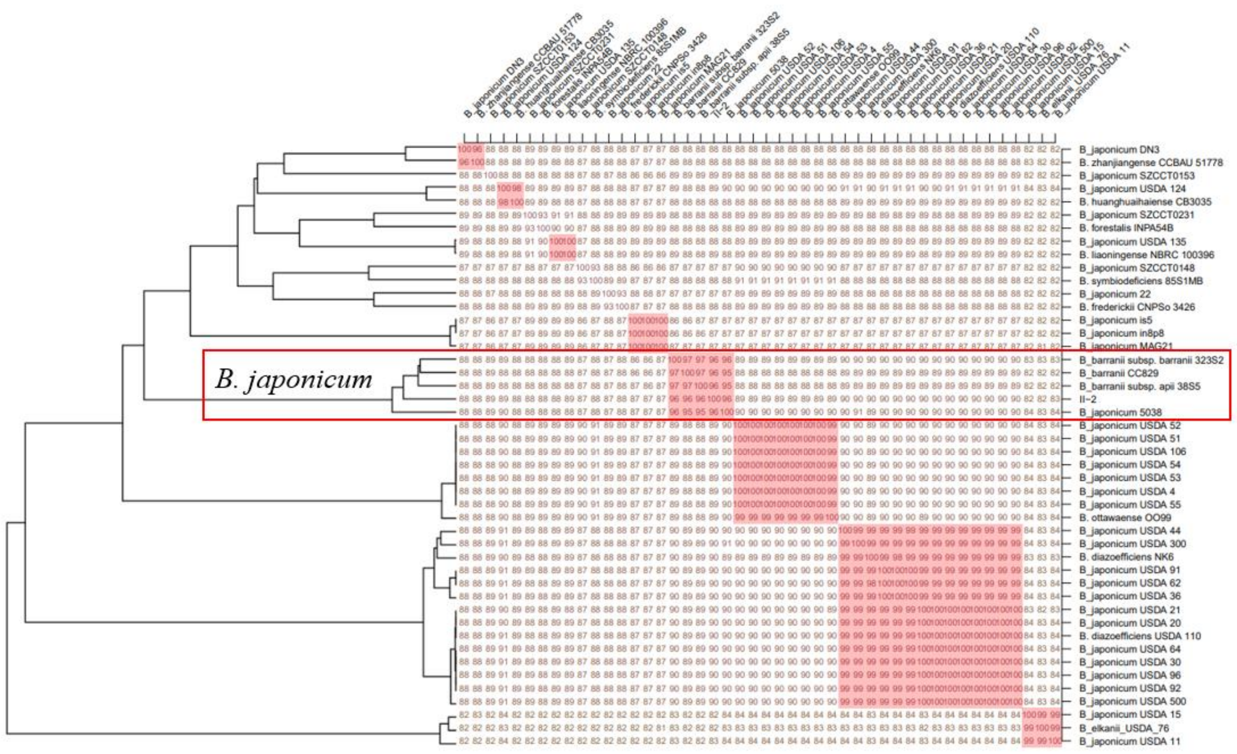

Figure S8. Matrices for comparison of the average nucleotide identity of 31 *B. japonicum* strains together with type strains of other *Bradyrhizobium* species.
